# Overexpression of *TaJAZ1* increases powdery mildew resistance through promoting reactive oxygen species accumulation in bread wheat

**DOI:** 10.1038/s41598-019-42177-y

**Published:** 2019-04-05

**Authors:** Yexing Jing, Jie Liu, Pan Liu, Dongfeng Ming, Jiaqiang Sun

**Affiliations:** 1 National Key Facility for Crop Gene Resources and Genetic Improvement, Institute of Crop Sciences, Chinese Academy of Agricultural Sciences, Beijing, 100081 China; 2 College of Life Science, Zaozhuang University, Zaozhuang, 277160 China

## Abstract

Powdery mildew, caused by the biotrophic fungal pathogen *Blumeria graminis* f. sp. *tritici*, is a major limitation for wheat yield. However, the molecular mechanisms underlying wheat resistance against powdery mildew remain largely unclear. In this study, we report the role of JASMONATE-ZIM domain protein TaJAZ1 in regulating bread wheat resistance against powdery mildew. We generated transgenic bread wheat lines over-expressing the truncated TaJAZ1 without the Jas motif, which showed increased *TaPR1/2* gene expression and reactive oxygen species accumulation, leading to enhanced resistance against powdery mildew. Simultaneously, we identified a Jasmonic acid (JA)-induced bHLH transcription factor TaMYC4 in bread wheat. We demonstrated that TaJAZ1 directly interacts with TaMYC4 to repress its transcriptional activity. Meanwhile, we show that the ZIM domain of TaJAZ1 interacts with the C terminus of TaNINJA, whereas the N-terminal EAR motif of TaNINJA interacts with the transcriptional co-repressor TaTPL. Collectively, our work pinpoints *TaJAZ1* as a favorable gene to enhance bread wheat resistance toward powdery mildew, and provides a molecular framework for JA signaling in bread wheat.

## Introduction

Bread wheat (*Triticum aestivum*; 2*n* = 42; AABBDD) is a major staple crop worldwide. In bread wheat, powdery mildew is caused by *Blumeria graminis* f. sp. *tritici* (*Bgt*), which is one of the most destructive fungal pathogens worldwide^1^. Breeding bread wheat varieties adapted to biotic and abiotic stresses are important agronomic traits. To improve bread wheat resistance against powdery mildew, it is useful to identify the key regulators for bread wheat defense responses. A previous study reported that simultaneous editing of all three *TaMLO* homoeologous in bread wheat confers heritable broad-spectrum resistance to powdery mildew^2^. We previously reported that ethylene signaling negatively regulates basal disease resistance against *Bgt*^3^.

The phytohormone Jasmonic acid (JA) and its derivatives are key regulators of multiple plant growth responses, including the response to biotic and abiotic stresses, as well as the developmental processes such as root growth and pollen development^4–13^. In the past decades, major discoveries in the model plant *Arabidopsis thaliana* have revealed a core JA signaling framework consisting of an F-box protein CORONATIN INSENSITIVE 1 (COI1), which forms the functional Skp-Cullin-F-box (SCF) E3 ubiquitin ligase complex SCF^COI1^ with Cullin1 and *Arabidopsis* Skp1-like protein ASK1^14–16^; a basic helix-loop-helix (bHLH) transcription factor (TF) MYC2 that acts as a master regulator in JA signaling pathway^17–19^; Jasmonate-ZIM domain (JAZ) proteins directly interact with MYC2 and repress its transcriptional activity^20–22^; JAZs that recruit the TOPLESS (TPL) co-repressor, either directly or through the NOVEL INTERACTOR OF JAZ (NINJA) adapter protein, which epigenetically inhibits expression of TF target genes^21^. In response to internal or external stimuli, elevated levels of JA-Ile promote SCF^COI1^-dependent degradation of the transcriptional repressors JAZs, thereby leading to the liberation of MYC2 from co-repressor TPL and subsequent induction of JA-responsive genes expression^20–24^. In addition, some components of JA signaling in rice (*Oryza sativa*) have been studied. One hundred and sixty-seven bHLH genes in the rice genome have been identified and defied as different subfamilies^25^. Three *OsFCL* (F-box AtCOI1-like protein) were predicted in the rice genome based on sequence comparison to *AtCOI1*.Twelve members of rice homologs of JAZ proteins were also selected including two conserved domains^26^. It has been demonstrated that OsMYC2 interacted with some OsJAZ proteins and regulated the expression of early JA-responsive genes to influence the bacterial blight resistance in rice^27^. Although the JA signaling pathways in *Arabidopsis* and rice have been well documented, the biological roles of JA and its signaling pathway in bread wheat remain largely unclear.

In this study, we demonstrated that the transgenic bread wheat lines over-expressing the truncated TaJAZ1 protein lacking the Jas motif displayed increased *TaPR1/2* gene expression and reactive oxygen species (ROS) accumulation, leading to enhanced resistance against powdery mildew. Simultaneously, we identified a jasmonate-induced bHLH transcription factor TaMYC4 in bread wheat. We demonstrate that TaJAZ1 interacts with TaMYC4 to repress its transcriptional activity. Collectively, our work demonstrates that TaJAZ1 positively regulates bread wheat resistance against powdery mildew, and provides a molecular framework for JA signaling in bread wheat.

## Results

### Identification and molecular characterization of bread wheat TaJAZ1

**Figure 1:**
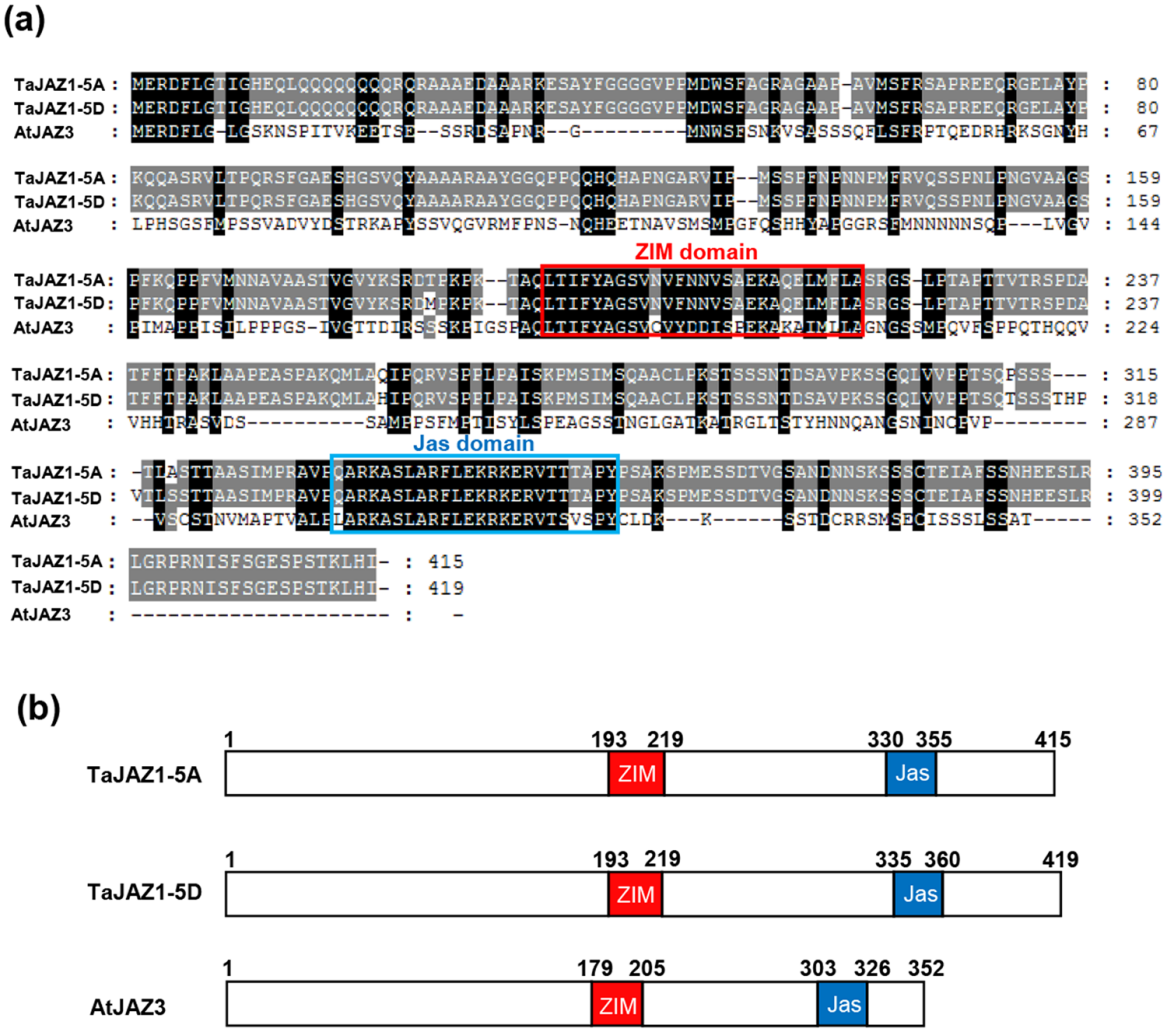
Molecular characterization of TaJAZ1 in bread wheat. (**a**) Amino acid sequences of TaJAZ1 and AtJAZ3 (At3g17860). TaJAZ1-5A and TaJAZ1-5D, respectively, represent TaJAZ1 proteins encoded by *TaJAZ1* genes from the bread wheat A and D subgenomes. The conserved ZIM domain and Jas domain were separately marked with the red and blue frames. The black and gray shade represent similarities. (**b**) The schematic representations of the protein structures of TaJAZ1-5A, TaJAZ1-5D and AtJAZ3.

In this study, to evaluate the biological roles of JAZs proteins in bread wheat, a BLAST search using Arabidopsis AtJAZ3 protein sequence identified two homologous sequences of 74% similarity. The two sequences, TaJAZ1-5A and TaJAZ1-5D, were located on *Triticum urartu* (AA) and *Aegilops tauschii* (DD) which are the two diploid progenitors of *Triticum aetivum* (bread wheat) (Supplementary Fig. S1)^28–30^. The predicted TaJAZ1-5A and TaJAZ1-5D proteins have 415 and 419 amino acids, respectively (Fig. 1a). A sequence alignment with the *Arabidopsis* JAZ3 protein revealed that TaJAZ1 shares the common structural features of AtJAZ3, containing conserved ZIM and Jas domains (Fig. 1b).

**Figure 2:**
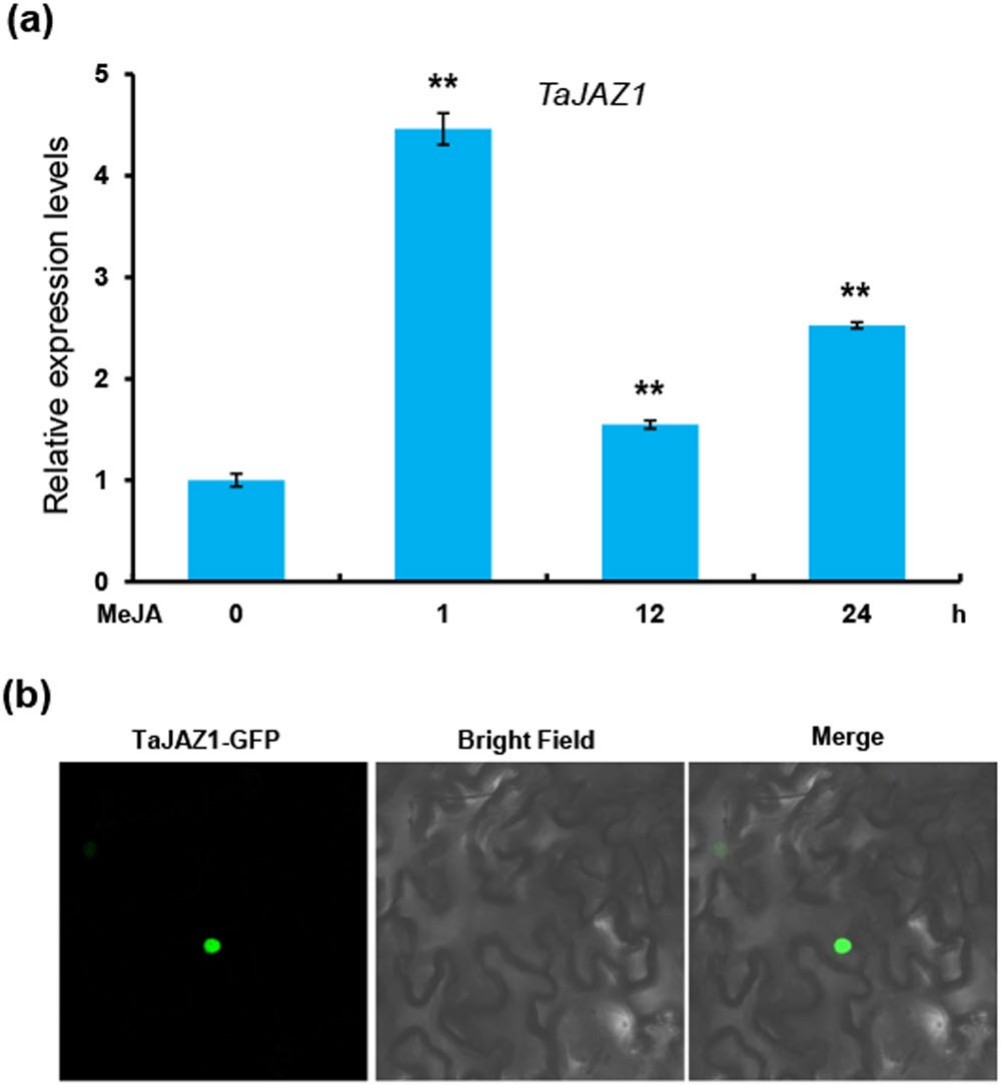
Expression pattern of *TaJAZ1* in response to JA treatment and subcellular localization of TaJAZ1. (**a**) qRT-PCR showing the induction of *TaJAZ1* by JA treatment. Samples were collected from KN199 treated with MeJA at different time points. The relative transcript levels of *TaJAZ1* were quantified by qRT-PCR and normalized against *TaGAPDH*. Error bars denote ± SD (n = 3). **P < 0.01 (Student’s *t* test). (**b**) Subcellular localization of TaJAZ1. The *35 S:TaJAZ1-GFP* was expressed in *N*. *benthamiana* leaves. GFP signal was detected 48 h post infiltration. The experiment was performed independently three times with similar results.

To investigate whether TaJAZ1 is involved in JA signaling pathway, we analyzed its expression pattern in response to exogenous JA treatment. Quantitative real time PCR (qRT-PCR) showed that the transcript levels of *TaJAZ1* were significantly increased in shortly (1–24 hpi) after JA treatment (Fig. 2a), the highest expression occurring one hour after JA treatment. This indicated that TaJAZ1 may be involved in JA signaling pathway in bread wheat.

To further determine the subcellular localization of TaJAZ1 in plant cells, *35S:TaJAZ1-GFP* was transiently expressed in *Nicotiana benthamiana* leaves. As shown in Fig. 2b, the fluorescence signal of GFP could be observed exclusively in the nuclei of *N*.*benthamiana* epidermal cells, confirming that TaJAZ1 was a nucleus-localized protein.

### Overexpression of *TaJAZ1ΔJas* results in enhanced bread wheat resistance against powdery mildew

**Figure 3:**
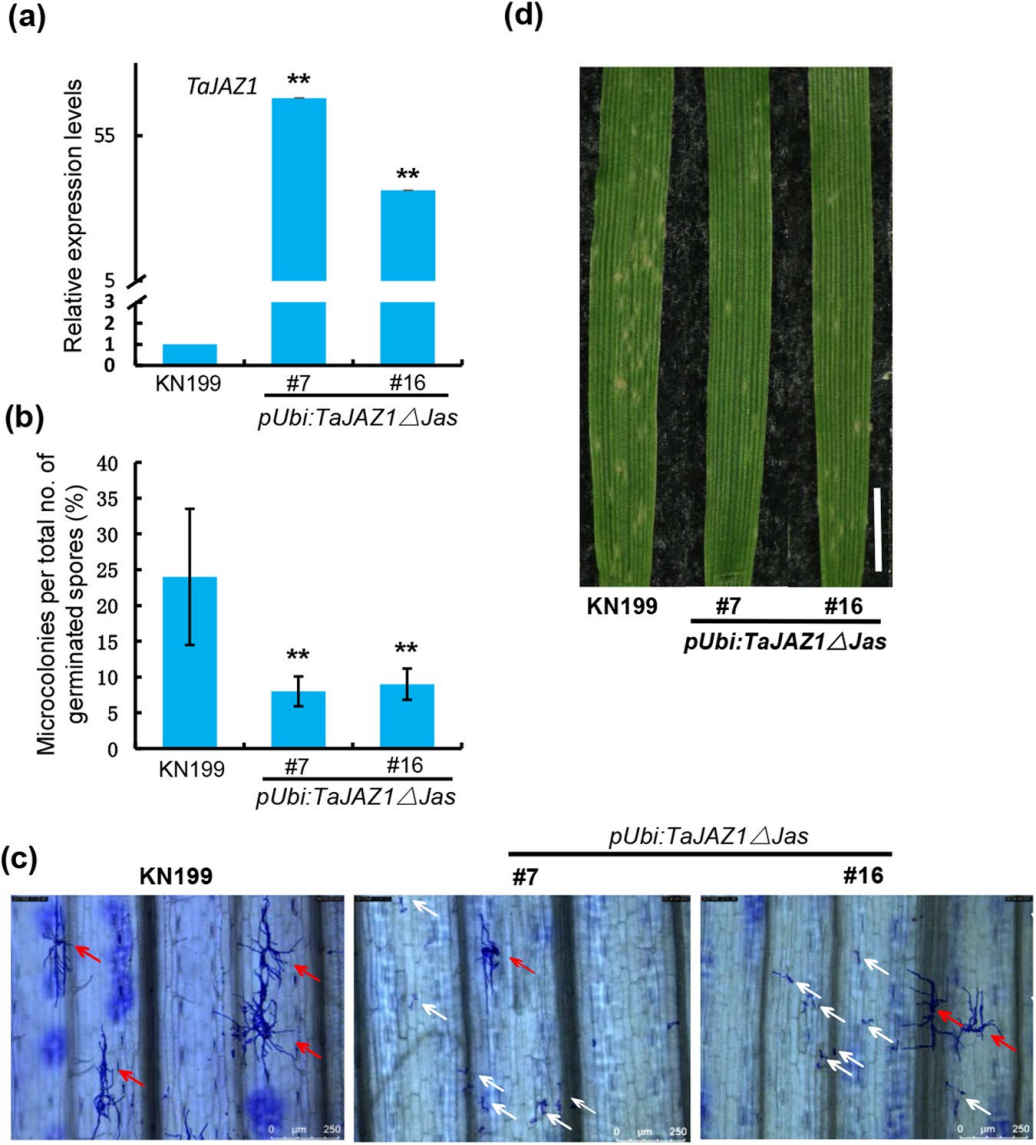
Overexpression of *TaJAZ1ΔJas* enhances the disease resistance of bread wheat against *Bgt*. (**a**) Relative expression levels of *TaJAZ1* in WT and two independent *pUbi:TaJAZ1ΔJas* transgenic lines. The expression levels of *TaJAZ1* were determined by qRT-PCR. Expression levels were normalized against *TaGAPDH*. #7 and #16 represent independent transgenic lines. (**b**) Statistical analysis of *B*. *graminis* MI% on bread wheat leaves of KN199 and two *pUbi:TaJAZ1ΔJas* transgenic lines. For each treatment, 7 to 15 leaves (3–4 cm in length) were analyzed independently. Spores of *B*. *graminis* on the leaves that form or did not form colonies were counted separately. MI% means the percentage of the successfully colonized *B*. *graminis* out of all analyzed spores. Error bars denote ± SD (n = 3). **P < 0.01 (Student’s *t* test). (**c**) *B*. *graminis* microcolony formation on the bread wheat leaves of KN199 and independent *pUbi:TaJAZ1ΔJas* transgenic lines. Samples were collected at 72 hpi and stained. Then leaves were observed microscopically and analyzed. Red arrows point to the successfully colonized *B*. *graminis*; white arrows point to the spores that germinated but did not form colony. Bars = 250 μm. (**d**) Macroscopic infection phenotypes of KN199 and transgenic lines. Representative leaves were removed and photographed at 7 d post inoculation. Bar = 1 cm.

In *Arabidopsis*, JAZ proteins were found to interact with COI1 mediate Jas domain leading to the degradation of JAZ proteins. To elucidate the biological roles of *TaJAZ1* in bread wheat, we generated transgenic bread wheat plants over-expressing truncated TaJAZ1, a stabilized form of TaJAZ1, in KN199 background^20,22^. The results of the qRT-PCR assay confirmed that *TaJAZ1ΔJas* was over-expressed in independent *pUbi:TaJAZ1ΔJas* transgenic lines (Fig. 3a). In order to test whether TaJAZ1 plays a role in regulating bread wheat resistance against powdery mildew, microcolony formation index (MI%) was calculated to evaluate the susceptibility of transgenic lines to *Bgt* at the seedling stage. In control samples, the MI% was around 20% to 30%, while in transgenic plants, the MI% were all significantly decreased to about 8% to 10% (Fig. 3b,c). After inoculation, we also observed a large number of visible conidia that were produced on the leaves at the seedling stage, although no obvious fungal growth was observed on the leaves of transgenic lines (Fig. 3d). These results illustrate an increased resistance to powdery mildew in *pUbi:TaJAZ1ΔJas* transgenic lines.

### Overexpression of *TaJAZ1ΔJas* results in up-regulation of *TaPR1/2* expression and ROS accumulation

**Figure 4:**
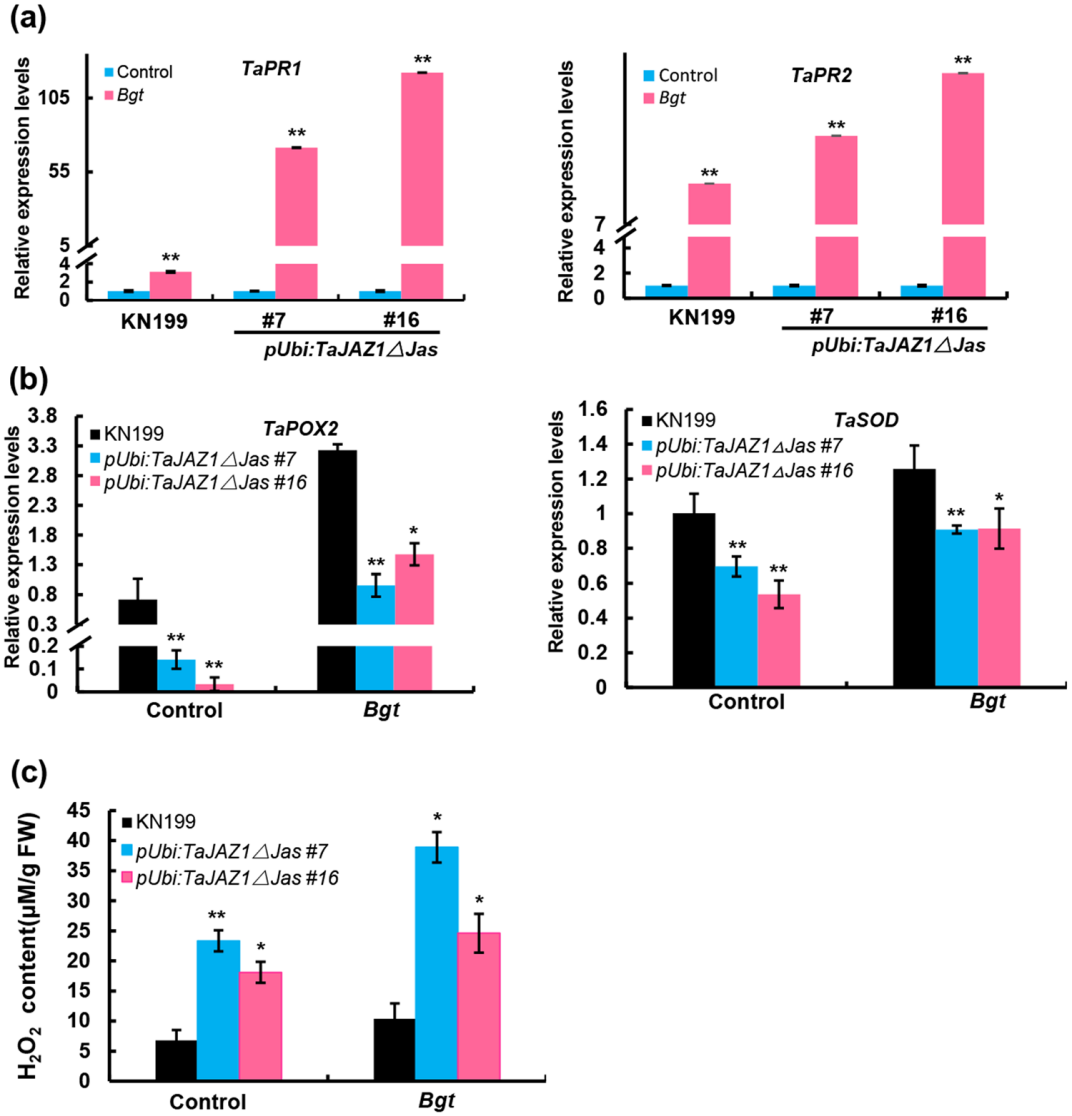
Over-expression of *TaJAZ1ΔJas* results in up-regulation of *TaPR1/2* expression and ROS accumulation in response to *Bgt*. (**a**) qRT-PCR analysis showing the expression levels of *TaPR1* and *TaPR2* in the *Bgt*-infected bread wheat leaves of KN199 and independent *pUbi:TaJAZ1ΔJas* transgenic lines. (**b**) qRT-PCR analysis showing the expression levels of *TaPOX2* and *TaSOD* in the *Bgt*-infected bread wheat leaves of KN199 and *pUbi:TaJAZ1ΔJas* transgenic lines. (**c**) H_2_O_2_ contents in the bread wheat leaves of KN199 and independent *pUbi:TaJAZ1ΔJas* transgenic lines treated with *Bgt*. Samples were collected at 24 hpi. Error bars denote ± SD (n = 3). Asterisks (** and *) above the bars represent significant differences between the control and each treatment at *P* < 0.01 and *P* < 0.05, respectively (Student’s *t* test).

We previously reported that the expression of pathogenesis-related (*PR*) genes *TaPR1* and *TaPR2* have been implicated in bread wheat resistance against powdery mildew^3^. To prove the association, we analyzed the expression patterns of *TaPR1* and *TaPR2* in response to the powdery mildew inoculation at 72 h post inoculation (hpi). As expected, we observed higher induction of the transcript levels of *TaPR1* and *TaPR2* in two independent *pUbi:TaJAZ1ΔJas* transgenic lines than that in KN199 upon the inoculation with *Bgt* (Fig. 4a).

Upon successful pathogens recognition, plants produce reactive oxygen species (ROS) including superoxide and its dismutation product hydrogen peroxide (H_2_O_2_)^31^. To detect the accumulation of ROS, we first examined the transcript levels of two ROS scavenging genes, *TaPOX2* and *TaSOD* in KN199 and two independent *pUbi:TaJAZ1ΔJas* transgenic lines at 12 hpi. The results showed that the expression levels of *TaPOX2* and *TaSOD* were significantly reduced in transgenic lines compared to KN199 either before or after the *Bgt* inoculation (Fig. 4b). We further analyzed the H_2_O_2_ levels in bread wheat leaves in response to *Bgt* infection. The results showed that the H_2_O_2_ levels were much higher in transgenic lines at 24 hpi compared to KN199 (Fig. 4c).

In summary, our results demonstrate that over-expression of *TaJAZ1ΔJas* enhances bread wheat resistance against powdery mildew, at least in part, through the up-regulation of *TaPR1/2* expression and ROS accumulation.

### Molecular characterization of the bHLH transcription factor TaMYC4

In *Arabidopsis*, the basic helix-loop-helix (bHLH) transcription factor AtMYC2 acts as a master regulator in JA signaling pathway^17,18,32^. To further elucidate the molecular mechanism by which TaJAZ1 reduces bread wheat susceptibility to powdery mildew, we used AtMYC2 protein sequence and isolated one homologous sequence of 41% similarity named *TaMYC4* on chromosome 1BL (Supplementary Fig. S2)^28–30,33^.

**Figure 5:**
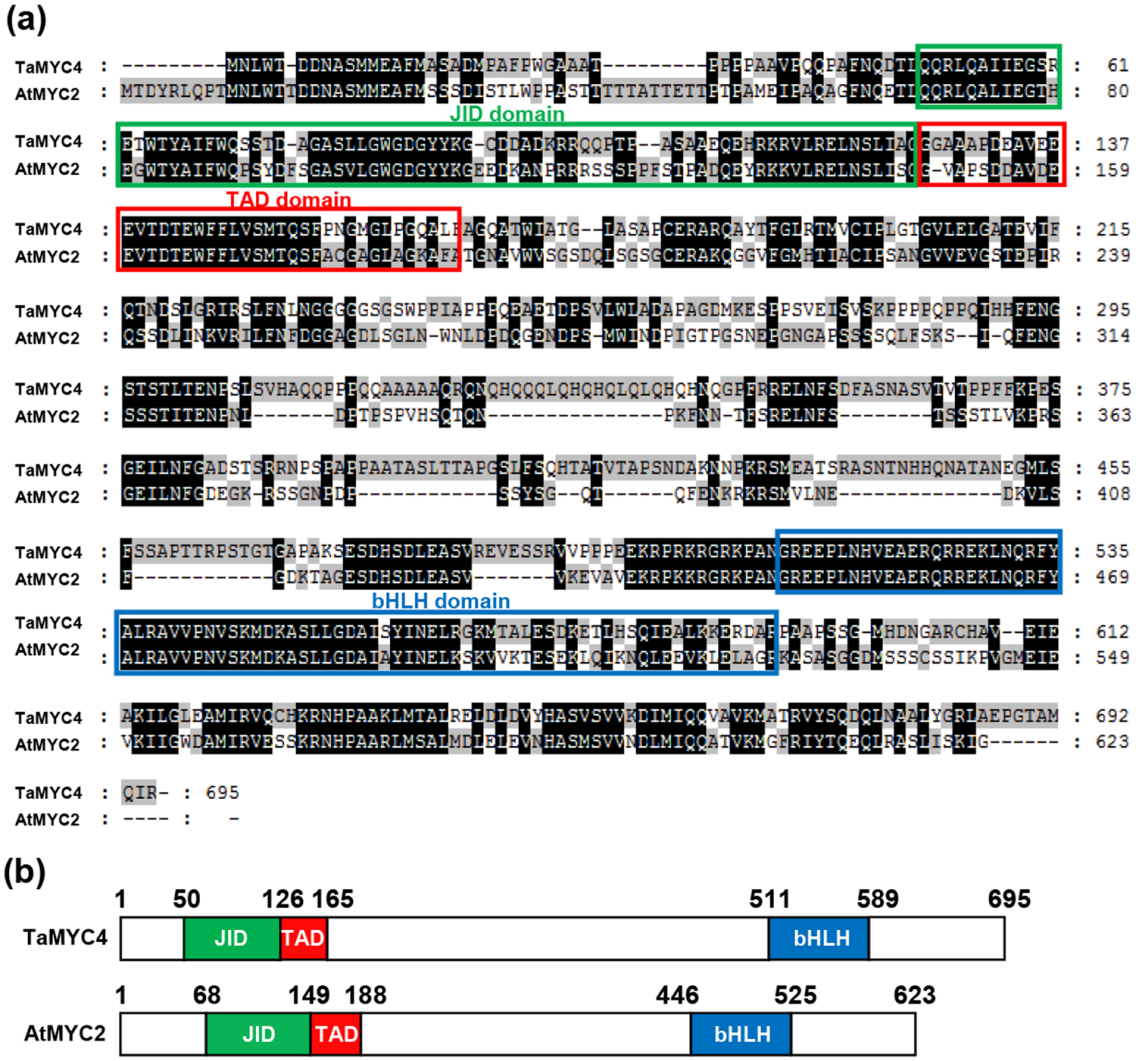
Molecular characterization of TaMYC4 in bread wheat. (**a**) Sequence alignment of TaMYC4 and AtMYC2 proteins. Green, red and blue frames separately represent the JID, TAD and bHLH domains of the two proteins. Residues that are not similar between the two proteins are shaded in gray or white. (**b**) The schematic representations of the protein structures of TaMYC4 and AtMYC2.

TaMYC4 shares the common structural features of AtMYC2, containing a JAZ Interaction domain (JID), a transcriptional activation domain (TAD) and a bHLH domain required for heterodimerization and binding to the G-box sequence in target promoters (Fig. 5a,b).

### TaJAZ1 interacts with TaMYC4

**Figure 6:**
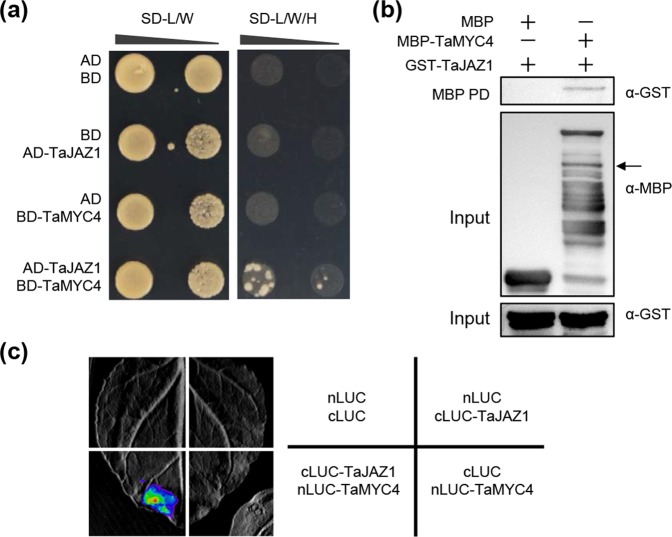
TaJAZ1 directly interacts with TaMYC4. (**a**) Yeast two-hybrid assay showing that TaJAZ1 physically interacts with TaMYC4. AD-TaJAZ1 and BD-TaMYC4 were co-expressed in yeast AH109 cells. The combinations of AD/BD-TaMYC4, BD/AD-TaJAZ1 and AD/BD were employed as negative controls. SD-L/W represents synthetic dextrose medium lacking Leu and Trp; SD-L/W/H means synthetic dextrose medium lacking Leu, Trp and His. (**b**) Pull-down assay showing the interaction between TaJAZ1 and TaMYC4. GST-TaJAZ1 protein was pulled down by MBP-TaMYC4 protein, and detected using an anti-GST antibody. MBP and MBP-TaMYC4 were detected using anti-MBP antibody. Arrow indicates specific band. Full-length blot is presented in Supplementary Fig. S3. (**c**) LCI assay showing the interaction between TaJAZ1 and TaMYC4 in *N*. *benthamiana* leaves. The images were collected 48 h after infiltration.

As described above, JID domain of TaMYC4 shares amino acid sequence homology with AtMYC2. This finding prompted us to test whether TaMYC4 is one target transcription factor of TaJAZ1. Thus, we investigated the physical interaction between TaJAZ1 and TaMYC4. First, we performed yeast two-hybrid (Y2H) assays by co-transforming TaJAZ1-AD and TaMYC4-BD into AH109 yeast (*Saccharomyces cerevisiae*) cells. As shown in Fig. 6a, the interaction was indeed observed between TaJAZ1 and TaMYC4. Then we conducted pull-down assays, and found that TaJAZ1 proteins were exclusively pulled down by MBP-TaMYC4, but not MBP (Fig. 6b). This demonstrated the direct interaction between TaMYC4 and TaJAZ1 *in vitro*. To further verify this interaction *in planta*, we carried out firefly luciferase complementation imaging (LCI) assays in *N*. *benthamiana* leaves^34^. As a result, strong fluorescence signals were observed exclusively in nLUC-TaMYC4 and cLUC-TaJAZ1 co-expressed samples, but not in the negative controls (Fig. 6c). Together, these data strongly demonstrate that TaMYC4 indeed interacts with TaJAZ1.

**Figure 7:**
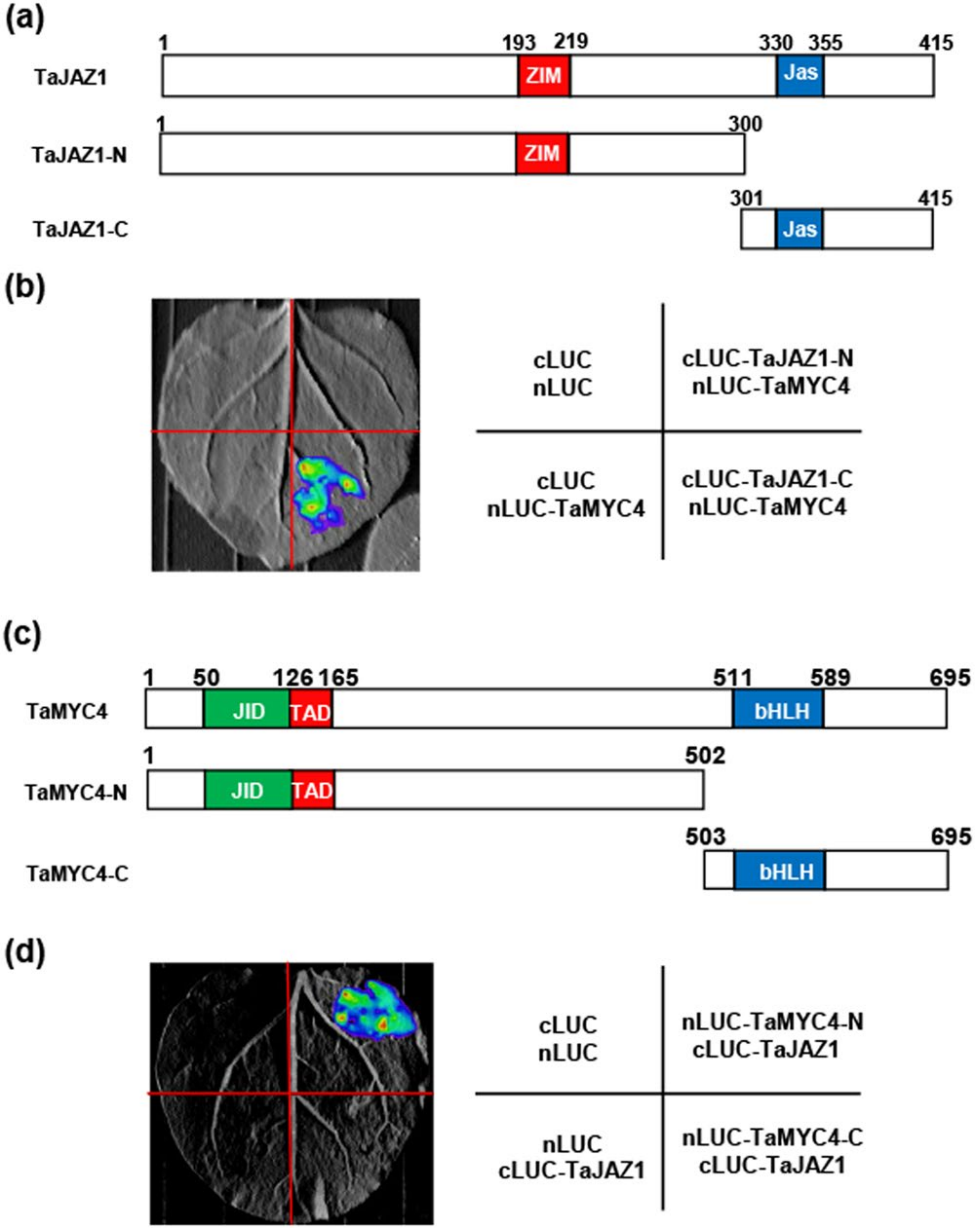
Mapping of the interaction domains of TaJAZ1 and TaMYC4. (**a**) Truncated versions of TaJAZ1 protein used for LCI assays. The N-terminus of TaJAZ1 (TaJAZ1-N) containing the ZIM domain represents the1–300 amino acids, and the C-terminus of TaJAZ1 (TaJAZ1-C) representing the 301–415 amino acids, contains the Jas domain. (**b**) LCI assays showing that the C-terminal part of TaJAZ1 mediated the interaction with TaMYC4. TaJAZ1-N and TaJAZ1-C were fused with cLUC and co-expressed with TaMYC4-nLUC in *N*. *benthamiana* leaves, and the interaction signals were collected 48 h after infiltration. (**c**) The truncated versions of TaMYC4 used for the transcriptional activation assay. TaMYC4-N, 1–502 aa; TaMYC4-C, 503–695 aa. (**d**) LCI assays showing that the N terminus of TaMYC4 mediated the interaction with TaJAZ1. The N- or C- terminal parts of TaMYC4 were fused with nLUC and co-expressed with TaJAZ1-cLUC. The signals were collected at 48 h after infiltration. Eight leaves were analyzed in each experiment, and three biological replications were performed.

To define the interaction domain of TaJAZ1 for TaMYC4 binding, we generated truncated TaJAZ1 derivatives, including the N-terminal and C-terminal parts of TaJAZ1, TaJAZ1-N (1–300 aa) and TaJAZ1-C (301–416 aa) (Fig. 7a). LCI assays in *N*. *benthamiana* confirmed that strong interaction signal was observed between TaJAZ1-C and TaMYC4, but not between TaJAZ1-N and TaMYC4 (Fig. 7b), suggesting that the C-terminal domain of TaJAZ1 is responsible for its interaction with TaMYC4.

Similarly, we used the LCI assays to map the interaction domain of TaMYC4 with TaJAZ1. Toward this goal, the full length TaMYC4 protein was divided into the N-terminal and C-terminal parts (Fig. 7c). As shown in Fig. 7d, the interaction was detected only between the N-terminal part of TaMYC4 and TaJAZ1.

### TaJAZ1 can repress the transcriptional activity of TaMYC4

**Figure 8:**
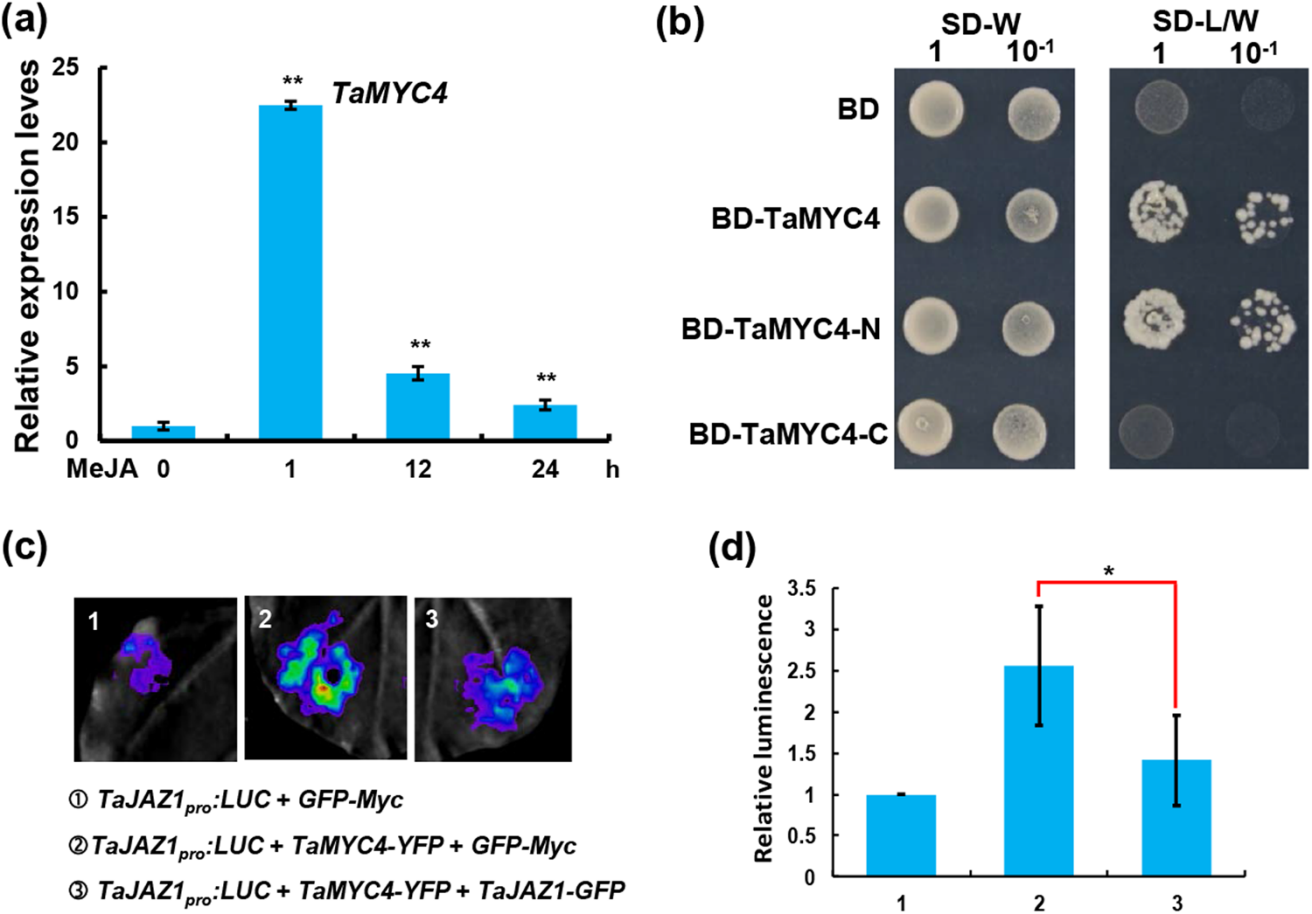
TaJAZ1 represses the transcriptional activation activity of TaMYC4. (**a**) MeJA-induced expression pattern of *TaMYC4*. Samples were collected from KN199 treated with MeJA at different time points. The relative transcript levels of *TaMYC4* were quantified by qRT-PCR and normalized against *TaGAPDH*. (**b**) Transcriptional activation assay for TaMYC4 in yeast cells. The full-length TaMYC4, TaMYC4-N and TaMYC4-C were expressed separately in yeast strain AH109. *pGBKT7* empty vector (EV) was used as a negative control. The transformed yeast cells were selected on synthetic dextrose medium lacking Trp (SD-W) and then transferred to synthetic dextrose medium lacking Leu and Trp (SD-L/W) with different dilution series (1 and 10^–1^). (**c**) Transient transcriptional activation assays showing that TaJAZ1 represses the transcriptional activation activity of TaMYC4. *N*. *benthamiana* leaves were infiltrated with three different combinations of *A*. *tumefaciens* strains containing indicated constructs. Images were taken at 48 h after infiltration. Ten independent *N*. *benthamiana* leaves were analyzed in each experiment, and totally three biological replications were carried out. (**d**) Quantification of luminescence intensities as shown in (**c**). Error bars denote ± SD (n = 3). *P < 0.05; **P < 0.01 (Student’s *t* test).

We further analyzed the expression pattern of *TaMYC4* in response to JA treatment. The results of the qPCR assay showed that the transcript levels of *TaMYC4* were quickly increased in response to JA treatment (Fig. 8a), suggesting that TaMYC4 may be involved in JA signaling in bread wheat.

Since TaMYC4 potentially acts as a bHLH transcription factor, we wondered whether it has transcriptional activation activity. To address this question, we performed the transcriptional activity assays in AH109 yeast (*Saccharomyces cerevisiae*) cells. As expected, the yeast cells harboring the full length TaMYC4 grew well on the selective medium as well as the yeast cells containing TaMYC4-N, suggesting that TaMYC4 could efficiently activate the transcription of the report gene acting as a transcriptional activation factor (Fig. 8b).

The above results already showed that the transcript levels of *TaJAZ1* were significantly increased in response to JA treatment (Fig. 3a). To further prove the relationship between TaJAZ1 and TaMYC4 in terms of transcriptional activity. We first cloned the *TaJAZ1* promoter (1932bp, Supplementary Fig. S4) and fused it with the *LUC* gene as a reporter. We carried out transient transcriptional activity assays in *N*. *benthamiana* leaves^34,35^. When *TaJAZ1*_*pro*_*:LUC* was co-infiltrated with *GFP-Myc* into *N*. *benthamiana* leaves, low level LUC activity was detected (Fig. 8c,d, coinfiltration 1). However, when *TaJAZ1*_*pro*_*:LUC* was co-expressed with *35S:TaMYC4*, the LUC activity was significantly elevated, which suggested that TaMYC4 could efficiently activate the transcription of *LUC* reporter gene (Fig. 8c,d, coinfiltration 2). More importantly, when *35S:TaJAZ1* was co-expressed with *TaJAZ1pro:LUC* and *35S:TaMYC4*, the TaMYC4-mediated activation of *TaJAZ1pro:LUC* was obviously suppressed (Fig. 8c,d, coinfiltration 3), which indicated that the transcriptional activation activity of TaMYC4 can be repressed by TaJAZ1.

### Molecular characterization of TaNINJA in bread wheat

We have shown that TaJAZ1 physically interacts with TaMYC4 to repress its transcriptional activity (Figs 6–8). But the molecular mechanism by which TaJAZ1 represses the transcriptional activity of TaMYC4 remains unknown. It has been reported that NOVEL INTERACTOR OF JAZ (NINJA) connects the co-repressor TOPLESS (TPL) to jasmonate signaling in *Arabidopsis*^21^. Therefore, we isolated one homologous sequence of 88% similarity with *AtNINJA* named as *TaNINJA* (Supplementary Fig. S5). BLAST result illustrated that *TaNINJA* was located on chromosomes 1A.

**Figure 9:**
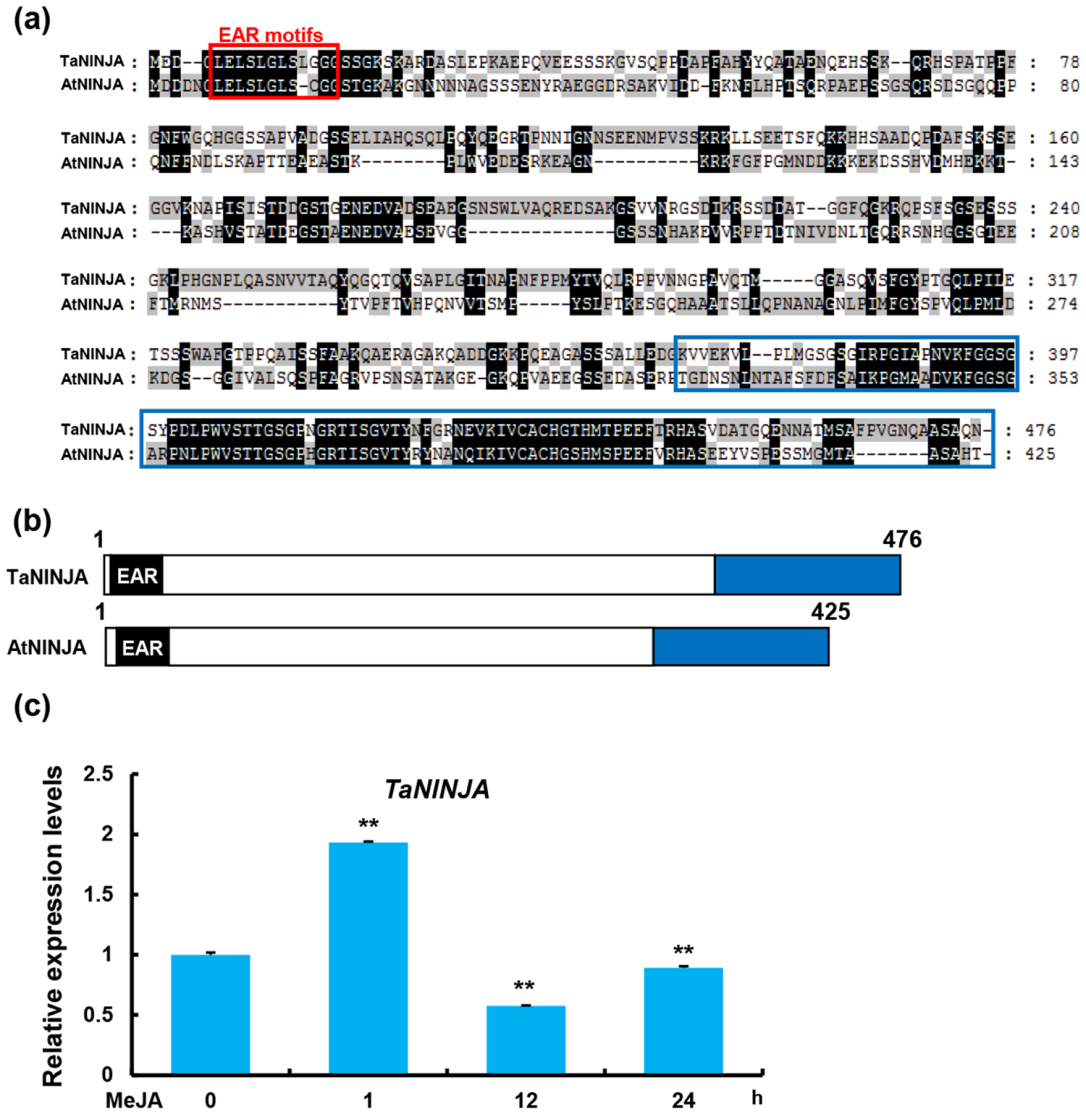
Molecular characterization of TaNINJA and MeJA-induced expression pattern of *TaNINJA*. (**a**) Sequence alignment of TaNINJA and AtNINJA proteins. Red frame represents the EAR motifs. Blue frame represents a conserved domain. Amino acid variations among TaNINJA and AtNINJA are marked by gray- and white-shaded background. (**b**) The box diagrams of the protein structures of TaNINJA and AtNINJA. (**c**) MeJA-induced expression pattern of *TaNINJA*. qRT-PCR were performed to determine the relative transcript levels of *TaNINJA* in the MeJA-treated KN199 leaves at different time points. The levels of *TaNINJA* were normalized against *TaGAPDH*. Error bars denote ± SD (n = 3) (**P < 0.01, Student’ s *t* test).

The sequence alignment showed that TaNINJA contains three ERF-associated amphiphilic repression (EAR) motifs (LXLXLX) at its N terminus and a conserved domain at the C terminus (Fig. 9a,b).

To test whether TaNINJA is associated with JA signaling in bread wheat, we analyzed the expression pattern of *TaNINJA* in response to JA treatment. The results of the qPCR assay showed that the transcript levels of *TaNINJA* were obviously up-regulated in shortly after JA treatment (Fig. 9c).

### TaNINJA tethers TaJAZ1 to the transcriptional co-repressor TaTPL

**Figure 10:**
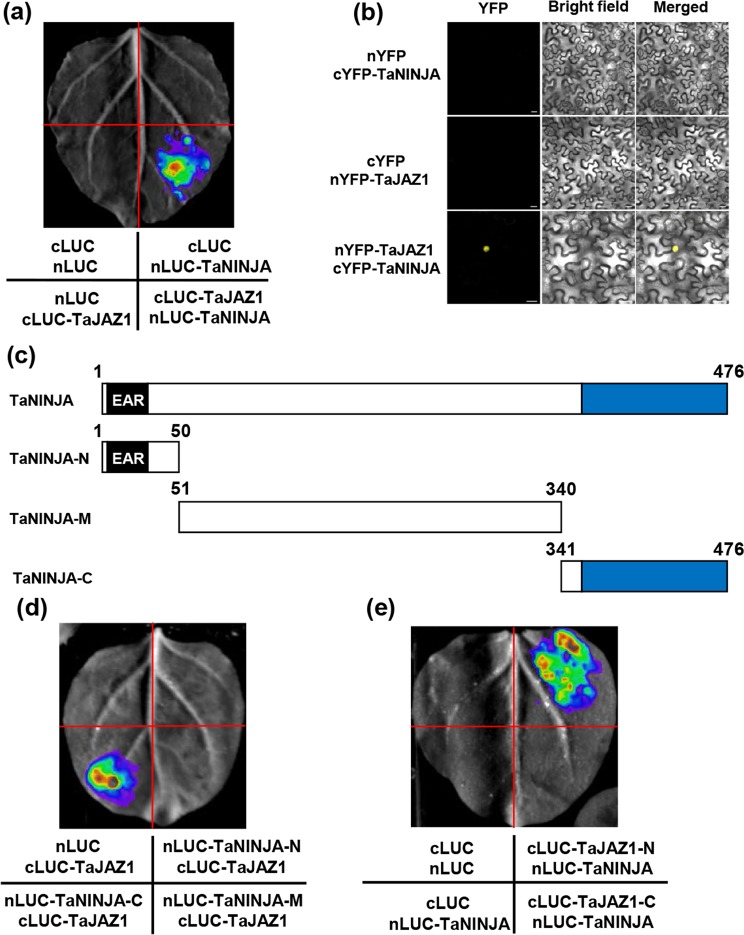
TaJAZ1 physically interacts with TaNINJA. (**a**) LCI assay showing the interaction between TaJAZ1 and TaNINJA. EV were used as negative controls. (**b**) BiFC assay illustrating the TaJAZ1-TaNINJA interaction in the nucleus of *N*. *benthamiana* leaf cells. TaJAZ1 and TaNINJA were separately fused with the N- and C-terminal part of YFP (nYFP and cYFP). YFP fluorescence was detected 48 h after infiltration. Bars = 20 µm. (**c**) Schematic representations of the domain structures of TaNINJA and truncated versions of TaNINJA proteins. (**d**) LCI assay illustrating that C-terminus of TaNINJA mediates its interaction with TaJAZ1. The nLUC and cLUC EVs were used as negative controls. (**e**) Mapping of the interaction domain of TaJAZ1 with TaNINJA. The signals of the interaction were collected 48 h after infiltration. Eight *N*. *benthamiana* leaves were analyzed in each experiment and similar results were observed.

To investigate whether TaNINJA links TaJAZ1 to the transcriptional co-repressor TaTPL for repression of TaMYC4 transcriptional activity, we conducted LCI assays in *N*. *benthamiana*. We fused TaNINJA to the N-terminal part of LUC to generate the nLUC-TaNINJA construct, and TaJAZ1 to the C-terminal part of LUC for cLUC-TaJAZ1. When nLUC-TaNINJA and cLUC-TaJAZ1 were co-infiltrated into *N*. *benthamiana* leaves, strong LUC activity was observed, whereas no LUC signal was detected in the negative controls (Fig. 10a). We also verified the interaction between TaNINJA and TaJAZ1 using bimolecular fluorescence complementation (BiFC) assay with a split yellow fluorescent protein (YFP) system in *N*. *benthamiana* leaves. As shown in Fig. 10b, strong fluorescence was exclusively detected in the nuclei of cells when cYFP-TaNINJA was transiently co-expressed with nYFP-TaJAZ1, but no fluorescence was detected in cells co-expressing nYFP/cYFP-TaNINJA or cYFP/nYFP-TaJAZ1. Together, these results suggest that TaNINJA interacts physically with TaJAZ1 in plant cells.

To define the interaction domains of TaNINJA and TaJAZ1, the full length TaNINJA protein was divided into the N-terminal, middle domain (MD) and C-terminal parts that fused with nLUC (Fig. 10c). The LCI assays showed that strong interaction signals were observed in the samples co-expressing nLUC-TaNINJA-C/cLUC-TaJAZ1 and nLUC-TaNINJA/cLUC-TaJAZ1-N, whereas no signal was detected in the control samples (Fig. 10d,e). These results suggest that the C-terminal parts of TaNINJA and TaJAZ1 mediate their physical interaction in plant cells.

**Figure 11:**
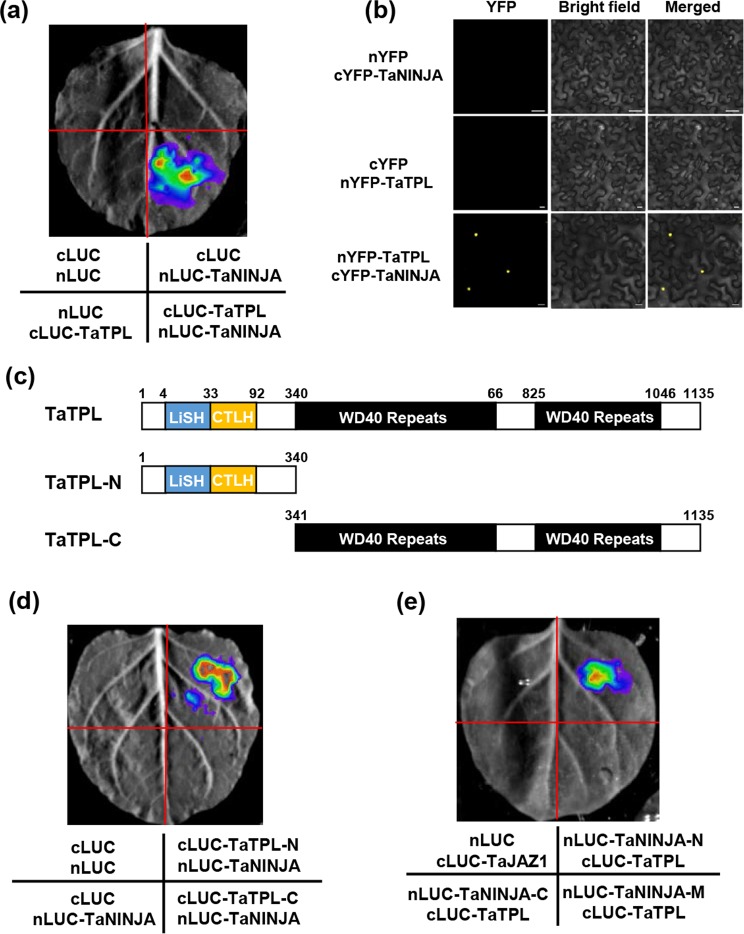
TaNINJA physically interacts with TaTPL. (**a**) LCI assays showing that TaNINJA interacts with TaTPL. TaNINJA and TaTPL were fused with nLUC and cLUC, respectively, and co-expressed in *N*. *benthamiana* leaves. (**b**) BiFC assay showing the interaction between TaNINJA and TaTPL. Bars = 20 µm. (**c**) Schematic representations of the domain structures of TaTPL and the truncated versions of TaTPL proteins. (**d**) A representative image of fluorescence signals in LCI assays for determination of the interaction domain of TaTPL with TaNINJA. (**e**) LCI assays showing that TaNINJA interacts with TaTPL mainly through N terminus of TaNINJA in *N*. *benthamiaona* leaves. The signals of the interaction were collected 48 h after infiltration. Eight *N*. *benthamiana* leaves were infiltrated in each experiment.

To test the possibility that TaJAZ1 recruits TaTPL through TaNINJA, we further analyzed the physically interaction between TaNINJA and TaTPL using LCI assay. We co-infiltrated nLUC-TaNINJA and cLUC-TaTPL into *N*. *benthamiana* leaves, strong LUC activity was observed (Fig. 11a), whereas no signal was detected in the negative controls. To further confirm the interaction, we conducted BiFC experiments. TaTPL and TaNINJA were separately fused with the N- and C- terminal parts of YFP to form nYFP-TaTPL and cYFP-TaNINJA, respectively. As a result, strong YFP fluorescent signal was observed exclusively from the nuclei of cells co-expressing nYFP-TaTPL and cYFP-TaNINJA, while the negative controls failed to yield any fluorescent signal (Fig. 11b).

In order to determine which domain of TaNINJA mediates the interaction with TaTPL, we performed the LCI assays. The results showed that strong fluorescent signal was only observed in the samples co-expressing the nLUC-TaNINJA-N and cLUC-TaTPL, whereas no signal was detected in negative controls (Fig. 11e). We next determined the domain of TaTPL responsible for the interaction with TaNINJA. The LCI assays showed that strong signals were only observed in the samples co-expressing cLUC-TaTPL-N and nLUC-TaNINJA, whereas no signal was detected in control samples (Fig. 11c,d). These results suggest that the N-terminal parts of TaNINJA and TaTPL mediate their physical interaction in plant cells.

## Discussion

Although some disease resistance genes have been applied in crops, their deployment is usually limited because of their negative effects on plant growth and development. For example, the barley *mlo* mutant causes early senescence-like phenotype^36^. Therefore, discovery of new disease resistance genes carrying no detrimental effects should be significant.

**Figure 12:**
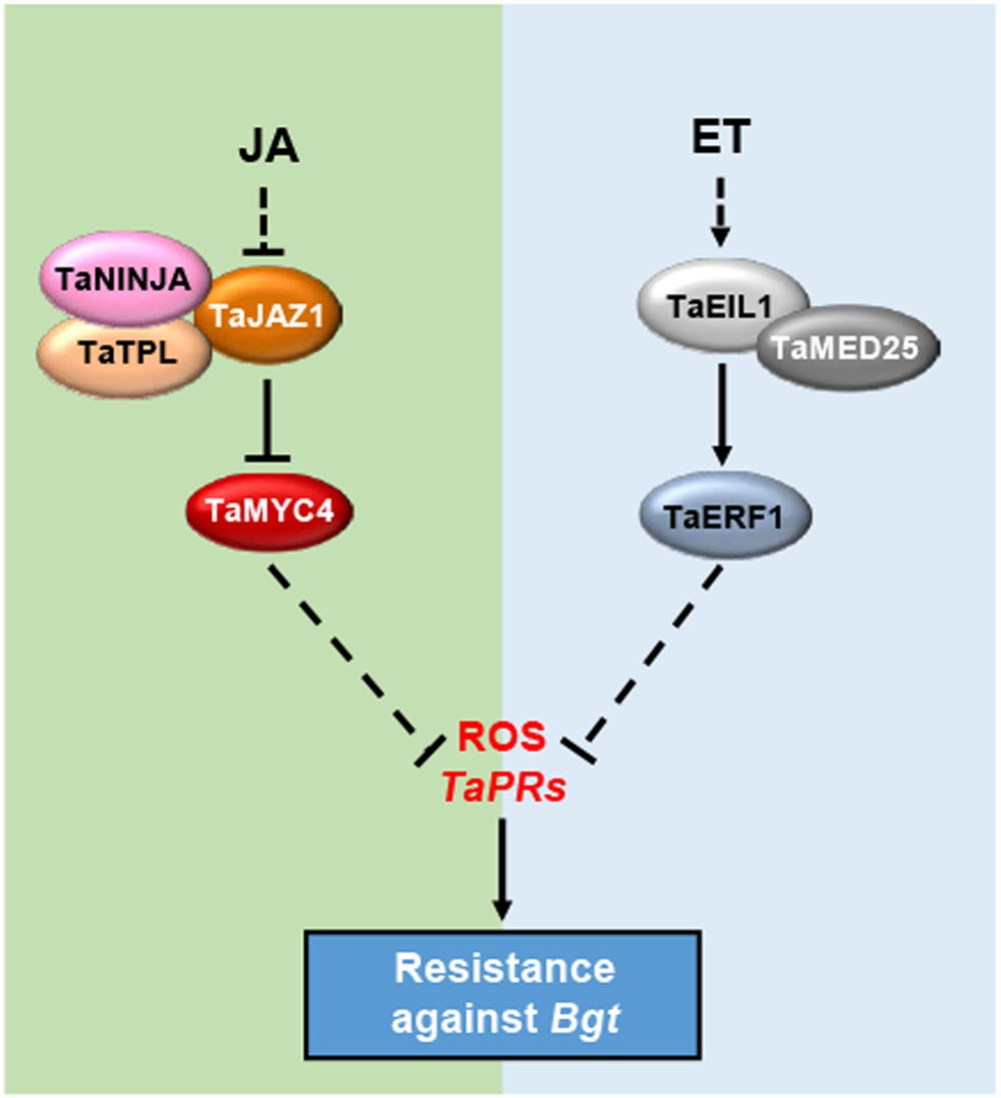
A proposed working model showing JA- and ET-mediated regulation of bread wheat resistance against powdery mildew. Under normal conditions, TaJAZ1, TaNINJA and TaTPL form a co-repressor complex that represses the transcriptional activity of TaMYC4. When JA accumulates in response to *Bgt* inoculation, TaJAZ1 is degraded to release TaMYC4 and other transcription factors, consequently leading to inhibition of ROS accumulation and *TaPRs* expression in bread wheat. Similarly, our previous study has shown that ET also inhibits ROS accumulation and *TaPRs* expression to negatively regulate wheat plant resistance against *Bgt*^3^.

Bread wheat is a major staple crop worldwide. Powdery mildew is often a destructive disease that results in significant loss of bread wheat productivity. Previous studies have shown that jasmonate is involved in the control of bread wheat responses to various biotic and abiotic stresses^37–40^. Wang *et al*. (2017) also identified fourteen *JAZ* genes in wheat based on the wheat genome data, and confirmed that some of these *JAZ* genes were transcriptionally affected by multifarious abiotic stress treatments and phytohormone^41^. However, the biological functions of jasmonate as well as these identified *JAZs* on wheat disease resistance, especially against *Bgt*, have yet to be elucidated. In this study, we not only focused on the function of one *JAZ* gene, *TaJAZ1*, but also showed the molecular mechanism by which *TaJAZ1* employed in regulating resistance against powdery mildew in bread wheat. We showed that over-expression of truncated TaJAZ1 lacking the Jas motif leads to enhanced defense gene expression and accumulation of ROS, resulting in increased resistance to powdery mildew (Figs 3b-d, 4a-c). Moreover, we characterized major components of JA signaling in bread wheat. This study elucidated core of the conserved JA signaling pathway in bread wheat that might be associated with powdery mildew resistance. Our model proposes that under normal conditions, TaNINJA directly links transcriptional repressor TaJAZ1 to the transcriptional co-repressor TaTPL for repression of the transcriptional activity of TaMYC4. After *Bgt* inoculation, TaJAZ1 is degraded and TaMYC4 is released to regulate resistance against *Bgt* through repressing ROS accumulation and *TaPRs* expression (Fig. 12).

Notably, different *JAZ* genes may have different effects on plant fertility. For example, in *Arabidopsis*, gain-of-function of JAZ1 leads to both JA-insensitivity and male sterility^22^, whereas gain-of-function of JAZ3 causes JA-insensitive phenotype, but not male sterility^20^. In this study, we showed that gain-of-function of TaJAZ1 conferred bread wheat disease resistance to powdery mildew, but caused no defects on plant growth phenotypes and fertility (Supplementary Figs S6 and S7). Therefore, we proposed that *TaJAZ1* is a favorable gene for improvement of bread wheat resistance against powdery mildew. Genome-editing technology such as CRISPR/Cas9 may be used to modify the Jas motif of specific *TaJAZ* genes, such as *TaJAZ1*, to create gain-of-function mutations for improvement of bread wheat resistance to powdery mildew. Moreover, it may be helpful to screen for natural elite alleles of *TaJAZ1* or other *TaJAZ* genes (e.g., with disrupted Jas motif), which might confer broad-spectrum resistance to powdery mildew. However, before we could utilize the *TaJAZ* genes to molecular breeding, one question should still be figured out, i.e. what kinds of effect may the *TaJAZ* genes have on the bread wheat resistance/susceptibility against other pathogens besides *Bgt*? Even though we observed an enhanced resistance against *Bgt* in the *pUbi:TaJAZ1ΔJas* transgenic wheat plants, we could not rule out the possibility that overdose of TaJAZ in wheat may also trigger susceptibility to some other pathogens (for example necrotrophic pathogens). Further work needs to be carried out to investigate the roles of *TaJAZ* genes on adult wheat resistance to powdery mildew as well as other important diseases in the field conditions.

In our previous study, we illustrated that both the Mediator subunit TaMED25 and the ethylene signaling activator TaEIL1 played negative roles in regulating bread wheat resistance against powdery mildew. The interaction between TaMED25 and TaEIL1 may recruit Pol II to the promoter of TaEIL1 target gene *TaERF1*, which further represses the accumulation of ROS by promoting the expression of some ROS-scavenging genes like *TaPOX2* and *TaADH*^3^. In this study, we showed that over-expression of the truncated jasmonate signaling repressor *TaJAZ1ΔJas* results in the accumulation of *Bgt*-triggered ROS as well as the up-regulation of *TaPRs* expression. These clues indicate that JA and ethylene signaling pathways may act synergistically in the regulation of bread wheat disease resistance against powdery mildew, probably through modulating the common downstream events, such as ROS accumulation and *PR*s expression (Fig. 12). The relation of TaMYC4 to the ROS accumulation in *TaJAZ1ΔJas* overexpression transgenic wheat plants is still not fully understood. Also unknown is whether TaMED25 can be employed to modulate the action of TaMYC4. Answers to these questions may be beneficial for the further understanding of the biological roles of JA signaling pathway in regulating bread wheat resistance against powdery mildew.

## Material and Methods

### Plant and fungal materials

The bread wheat cultivar Kenong 199 (KN199) and transgenic lines used for genes cloning, JA treatment and powdery mildew infection were grown in a growth chamber with a 16-h/8-h(22 °C) day/night cycle for seven days in healthy conditions. In the experimental field (39°96′N, 116°33′E) of the Institute of Crop Science, the Chinese Academy of Agricultural Sciences, Beijing, materials were planted in October 2017 and harvested in June the next year. *Nicotiana benthamiana* was grown in the greenhouse at 22 °C with a 16-h day/8-h night light photoperiod.

*Blumeria graminis* f. sp. *tritici* isolate E09 was maintained on bread wheat KN199, and kept at 12-h/12-h, 20 °C/18 °C day/night cycle in a spore-proof chamber. The inoculation experiments carried out the same conditions.

### DNA constructs and gene cloning

DNA constructs used in this study were built following classic molecular biology protocols and using Gateway (Invitrogen) technology. Genes were cloned by the homology cloning method. To find the homology proteins of *Arabidopsis thaliana* in bread wheat, BLAST was performed based on the database of *Triticum urartu* and *Aegilops tauschii*^28,29^. Then specific primers (Supplementary Table S2) were designed refer to the genome sequences of *T*. *urartu* and *A*. *tauschii* for the nested PCR to obtain the coding sequences (CDS) from bread wheat KN199 complementary DNA (cDNA). PCR products were cloned into pEasy-Blunt Cloning Kit (TransGen Biotech, CB101-02) for sequencing. All details of the DNA constructs are listed in Table S1. All primers used in the study are shown in Table S2.

### RNA extraction and gene expression analyses

For gene expression assays, the 7-d-old seedlings of WT KN199 and the transgenic lines were cultured in water and treated with 10 mM MeJA or powdery mildew. Samples were harvested at indicated time points. Total RNA was extracted using Trizol (Invitrogen) reagent, and cDNAs were obtained from 2 μg total RNA using 5× All-In One RT MasterMix system (Applied Biological Materials). Quantitative reverse transcription-polymerase chain reaction (qRT-PCR) was performed with SYBR^®^Premix Ex Taq Kit (TaKaRa). The transcriptional expression levels of target genes were normalized to *TaGAPDH*. All primers for qRT-PCR assays are listed in Table S2.

### Subcellular localization analyses

Full-length CDS of *TaJAZ1* was cloned into pGWB5 vector to fuse with the green fluorescent protein (GFP) gene sequence^42^. TaJAZ1-GFP was transiently expressed in *N*. *benthamiana* leaves, and the fluorescence signal of GFP was observed 48 h after infiltration.

### Powdery mildew infection and microcolony formation analyses

Powdery mildew infection and microscopic analyses were performed as reported previously with some modifications^3^. The inoculations for MI% calculation were performed with low density (5–10 conidia mm^−2^). The visible conidia observations were performed with high-density inoculations (20–50 conidia mm^−2^). Phenotypes of representative leaves of plants were taken 7 d after inoculation. Leaves from 7-d-old seedlings were infected with *Bgt* strain E09. Three days later, leaves were collected and fixed with ethanol:acetic acid solution (1:1, v/v), and then destained with lactoglycerol solution (lactic acid: glycerol:water, 1:1:1, v/v/v). After the staining with 0.1% (w/v) Coomassie Brilliant Blue R250, MI% was analyzed by microscope. Here, MI% represents the percentage of successfully colonized *B*. *graminis* out of all analyzed spores. In each replication, 7 to 15 independent leaves (3–4 cm in length) from 3 to 5 plants were analyzed, and the mean MI% values were calculated. Totally, three independent replications were conducted. The statistical significance was evaluated by Student’s *t* test.

### H_2_O_2_ measurement

Seven-d-old seedlings of KN199 and two *pUbi:TaJAZ1ΔJas* transgenic lines were treated with powdery mildew spores. Samples were harvested 24 h post inoculation (hpi). The content of H_2_O_2_ in the samples were analyzed as reported previously using the Amplex Red Hydrogen Peroxide assay kit (Invitrogen)^43^. First, samples were ground with liquid nitrogen, and mixed with 3 volumes of H_2_O. Then, samples were centrifugated at 12,000 rpm at 4 °C for 20 min, and the supernatants were used for the H_2_O_2_ assay.

### Yeast experiments

For transcriptional activation activity assays, the GAL4-BD derivatives were separately transformed into the yeast (*Saccharomyces cerevisiae*) strain AH109. Then the transformed yeast strains were grown on synthetic dextrose medium lacking Trp (SD-W), and then transferred to the SD medium lacking Leu and Trp (SD-L/W) for evaluation of the transcriptional activation activity.

For yeast two-hybrid analysis, *TaJAZ1* and *TaMYC4* were separately cloned into *pGADT7* and *pGBKT7*. The combination of constructs AD/BD, AD/BD-TaMYC4, BD/AD-TaJAZ1 and AD-TaJAZ1/BD-TaMYC4 were co-transformed into yeast. The transformed yeast strains were first grown on the SD-L/W medium, and then selected on SD medium lacking Leu, Trp and His (SD-L/W/H) for interaction analysis.

### Transactivation assays in *N*. *benthamiana* leaves

The transactivation assays were performed in *N*. *benthamiana* leaves as described previously^35^. About 2-kb *TaJAZ1* promoter was fused with the luciferase reporter gene *LUC* by Gateway reactions (Invitrogen) into the plant binary vector pGWB35 to generate the reporter construct *TaJAZ1*_*pro*_*:LUC*^42^. The full-length *TaJAZ1* and *TaMYC4* were separately cloned into the plant binary vector pGWB5 and pEarlyGate 101 to generate the effector constructs *35S:TaJAZ1-GFP* and *35S:TaMYC4-YFP*. Different combinations of reporters and effectors were coinfiltrated into *N*. *benthamiana*, and the LUC signals were observed and analyzed 48 h after infiltration by using Night SHADE LB 985 system (Berthold, Germany).

### Pull-down assay

The maltose binding protein (MBP) tagged TaMYC4 and glutathione S-transferase (GST) tagged TaJAZ1 proteins were expressed in *Escherichia coli* strain Transetta-DE3 (TransGen biotech, CD801) by induction with 0.5 mM isopropyl β-D-1-thiogalactopyranoside at 18 °C for 16 h. Cells were collected by centrifugation and then resuspended with the column buffer (for MBP-TaMYC4; 20 mM Tris-HCl, 0.2 M NaCl, 0.5 M EDTA, 1 mM PMSF, 1 mM DTT, 1 × cocktail). After sonication, samples were centrifuged at 4 °C for 30 min and the supernatant was used for further assays. Equal volumes of MBP and GST-TaJAZ1 or MBP-TaMYC4 and GST-TaJAZ1 were incubated with amylose resin beads, and analyzed by immunoblotting with anti-GST (Cat# CW0144, CWbiotech, Beijing, China) and anti-MBP (Cat# CW0288, 453CWbiotech, Beijing, China) antibodies.

### LCI assays

The LCI assays were performed in *N*. *benthamiana* leaves as described previously with modifications^44^. The firefly LUC enzyme was divided into the N-terminal part (nLUC) and the C-terminal part (cLUC). Different indicated plasmid pairs were transformed into *Agrobacteria* strain GV3101 and co-infiltrated into *N*. *benthamiana* leaves. After 48 h, the luciferase luminescence areas on the leaves were imaged using Night SHADE LB 985 system (Berthold, Germany).

### BiFC assays

The BiFC assays were carried out as described previously^45^. TaJAZ1 and TaNINJA was separately fused with the N-terminal and the C-terminal half of YFP. nYFP and cYFP derivative constructs were co-injected into *N*. *benthamiana* leaves. The YFP signals were visualized by using the confocal microscope (Carl Zeiss, LSM880) at 48 hpi.

## Supplementary information

Supplementary Info File

## Data Availability

These sequence data have been submitted to the GenBank databases under accession numbers: TaJAZ1-5A, MH063272; TaJAZ1-5D, MH063273; TaMYC4, MH063274; TaNINJA, MH063275. Addresses are as follows: www.ncbi.nlm.nih.gov/genbank.
