# Supplementary material for: Overexpression of TaJAZ1 increases powdery mildew resistance through promoting reactive oxygen species accumulation in bread wheat
Source: Sci Rep. 2019 Apr 5;9:5691. doi: 10.1038/s41598-019-42177-y (PMC6451029; doi:10.1038/s41598-019-42177-y)
Supplement: Supplementary file 1 — Supplementary Info File [file 41598_2019_42177_MOESM1_ESM.pdf]

Overexpression of *TaJAZ1* increases powdery mildew resistance through promoting reactive oxygen species accumulation in bread wheat

Yexing Jing[1,#], Jie Liu[1,#], Pan Liu[1,#], Dongfeng Ming[2] and Jiaqiang Sun[1,*]

Supplemental Figure 1

```
TaJAZ1-5A : ATGGAGAGGGACTTCCTGGGCACCATAGGCCACGAGCAGCTGCAGCAGCAGCAGCAGCAGCAGCGCCAGCGCGCCGC :   80
TaJAZ1-5D : ATGGAGAGGGACTTCCTGGGCACCATAGGCCACGAGCAGCTGCAGCAGCAGCAGCAGCAGCAGCGCCAGCGCGCCGC :   80

TaJAZ1-5A : CGCCGAGGACGCCGCCGCCAGAAAGGAGTCAGCTTACTTTGGGGGAGGAGGAGTGCCGCCCATGGATTGGTCCTTCGCTG :  160
TaJAZ1-5D : CGCCGAGGACGCCGCCGCCAGAAAGGAGTCAGCTTACTTTGGGGGAGGAGGAGTGCCGCCCATGGATTGGTCCTTCGCTG :  160

TaJAZ1-5A : GCAGGGCCGGGGCCGCGCCGGCGGTCATGTCCTTCAGGTCGGCGCCGAGGGAGGAGCAGCGGGGCGAGCTCGCCTACCCC :  240
TaJAZ1-5D : GCAGGGCCGGGGCCGCGCCGGCGGTCATGTCCTTCAGGTCGGCGCCGAGGGAGGAGCAGCGGGGCGAGCTCGCCTACCCC :  240

TaJAZ1-5A : AAGCAGCAGGCCTCCCGCGTCCTGACGCCACAGAGATCGTTTGGTGCTGAGAGCCACGGCAGCGTGCAGTACGCCGCCGC :  320
TaJAZ1-5D : AAGCAGCAGGCCTCCCGCGTCCTGACGCCACAGAGATCGTTTGGTGCTGAGAGCCACGGCAGCGTGCAGTACGCCGCCGC :  320

TaJAZ1-5A : CGCGCGTGCGGCTTACGGCGGGCAGCCTCCGCAGCAGCACCAGCATGCTCCTAATGGTGCTAGAGTGATTCCAATGTCGT :  400
TaJAZ1-5D : CGCGCGTGCGGCTTACGGCGGGCAGCCTCCGCAGCAGCACCAGCATGCTCCTAATGGTGCTAGAGTGATTCCAATGTCGT :  400

TaJAZ1-5A : CGCCGTTCAATCCCAACAATCCCATGTTCAGGGTTCAGAGTTCGCCTAACCTCCCGAACGGTGTTGCTGCTGGTAGCCCG :  480
TaJAZ1-5D : CGCCGTTCAATCCCAACAATCCCATGTTCAGGGTTCAGAGTTCGCCTAACCTCCCGAACGGTGTTGCTGCTGGTAGCCCG :  480

TaJAZ1-5A : TTCAAACAACCGCCTTTCGTGATGAACAATGCGGTGGCTGCTTCGACTGTTGGTGTCTATAAATCAAGGGACACGCCGAA :  560
TaJAZ1-5D : TTCAAACAACCGCCTTTCGTGATGAACAATGCGGTGGCTGCTTCGACTGTTGGTGTCTATAAATCAAGGGACATGCCAAA :  560

TaJAZ1-5A : GCCAAAGACAGCGCAATTAACTATCTTCTATGCTGGTTCTGTCAATGTATTCAACAACGTCTCAGCAGAAAAGGCTCAGG :  640
TaJAZ1-5D : GCCCAAGACAGCGCAGTTAACTATCTTCTACGCTGGTTCTGTCAATGTATTCAACAACGTCTCGGCAGAAAAGGCTCAGG :  640

TaJAZ1-5A : AGCTTATGTTCTTGGCTAGCAGAGGATCTCTTCCAACCGCACCCACTACTGTTACTCGCAGCCCAGATGCAACCTTTTTC :  720
TaJAZ1-5D : AGCTTATGTTCTTGGCTAGCAGAGGATCTCTTCCAACTGCACCCACTACTGTTACTCGCAGCCCAGATGCAACCTTTTTC :  720

TaJAZ1-5A : ACTCCGGCTAAACTCGCCGCCCCTGAGGCTTCACCTGCAAAGCAGATGCTAGCTCAGATACCACAGCGTGTTTCACCTCC :  800
TaJAZ1-5D : ACTCCCGCTAAACTCGCAGCACCTGAGGCTTCACCTGCAAAGCAGATGCTAGCTCACATACCACAGCGTGTTTCACCTCC :  800

TaJAZ1-5A : TTTGCCAGCCATTTCCAAACCGATGTCCATCATGTCTCAAGCTGCATGTCTCCCCAAGAGCACATCTAGCTCCAACACCG :  880
TaJAZ1-5D : TTTGCCGGCCATTTCCAAACCGATGTCCATCATGTCTCAAGCTGCATGTCTCCCCAAGAGCACATCTAGCTCCAACACCG :  880

TaJAZ1-5A : ATTCCGCAGTGCCAAAATCTTCAGGCCAGTTGGTTGTGCCTCCCACAAGTCAGCCCTCGTCGTCGAC------------A :  948
TaJAZ1-5D : ATTCCGCAGTGCCAAAATCTTCAGGCCAGTTGGTTGTGCCTCCCACAAGTCAGACCTCGTCGTCGACTCATCCTGTGACA :  960

TaJAZ1-5A : CTAGCGTCCACCACTGCAGCAAGTATTATGCCAAGAGCTGTTCCTCAAGCTCGGAAGGCATCCCTTGCCCGATTCTTGGA : 1028
TaJAZ1-5D : CTATCGTCCACCACTGCTGCAAGTATTATGCCAAGAGCTGTTCCTCAAGCACGTAAGGCATCCCTTGCCCGATTCTTGGA : 1040

TaJAZ1-5A : GAAAAGGAAAGAAAGGGTGACGACTACGGCGCCATATCCATCAGCCAAGAGCCCGATGGAGAGCAGCGACACGGTCGGAA : 1108
TaJAZ1-5D : GAAAAGGAAAGAAAGAGTGACGACTACGGCGCCATATCCATCAGCCAAGAGCCCGATGGAGAGCAGCGACACGGTCGGAA : 1120

TaJAZ1-5A : GCGCCAACGACAACAACAGCAAGTCCTCATCGTGCACAGAGATCGCCTTCTCAAGCAACCATGAAGAGTCGCTGCGCCTA : 1188
TaJAZ1-5D : GCGCCAACGACAACAACAGCAAGTCCTCGTCGTGCACGGAGATCGCCTTCTCAAGCAACCATGAAGAGTCGCTGCGCCTA : 1200

TaJAZ1-5A : GGCCGGCCCAGGAACATCAGCTTTAGCGGGGAGTCCCCGAGTACAAAATTACACATCTGA : 1248
TaJAZ1-5D : GGCCGGCCCAGGAACATCAGCTTTAGTGGGGAGTCCCCGAGTACAAAATTACACATCTGA : 1260
```

Supplemental Figure 1. Alignment of *TaJAZ1-5A* and *TaJAZ1-5D* coding sequences.
TaJAZ1-5A and *TaJAZ1-5D* represent *TaJAZ1* CDS derived from the A and D subgenomes of bread wheat cultivar KN199. Nucleotide variations among *TaJAZ1-5A* and *TaJAZ1-5D* are shaded in gray.

Supplemental Figure 2

TaMYC4-1B

5’-
ATGAACCTGTGGACGGACGACAACGCCTCCATGATGGAGGCCTTCATGGCCTCCGCCGAC
ATGCCGGCCTTCCCCTGGGGCGCTGCGGCCACCCCGCCGCCGCCGGCCGCCGTGCCGC
AGCAGCCGGCCTTCAACCAGGACACGCTGCAGCAGCGCCTGCAGGCCATCATCGAGGGC
TCCAGGGAGACCTGGACCTACGCCATCTTCTGGCAGTCCTCCACCGACGCCGGCGCCTC
GCTCCTCGGCTGGGGCGACGGCTACTACAAGGGCTGCGACGACGCCGACAAGCGCCGC
CAGCAGCCCACCCCGGCCTCCGCCGCCGAGCAGGAGCACCGCAAGCGCGTCCTCCGGG
AGCTCAACTCGCTCATAGCCGGGGGAGGCGCCGCCGCGCCCGACGAGGCCGTCGAGGA
GGAGGTCACGGACACCGAGTGGTTCTTCCTCGTCTCCATGACCCAGTCCTTCCCCAACGG
GATGGGCTTGCCGGGCCAGGCGCTCTTCGCCGGCCAGGCCACCTGGATCGCCACGGGG
CTCGCCAGCGCGCCCTGCGAGCGGGCCAGGCAGGCCTACACCTTCGGCCTCCGCACCAT
GGTCTGCATCCCCCTCGGCACCGGCGTGCTCGAGCTCGGCGCCACCGAGGTCATCTTCC
AGACCAACGATAGCTTGGGGAGGATCCGCTCGCTCTTCAACCTCAACGGCGGAGGAGGG
GGCTCTGGATCCTGGCCGCCCATCGCGCCGCCGCCGCAGGAGGCGGAGACGGATCCGT
CCGTGCTCTGGCTCGCCGACGCGCCGGCCGGGGACATGAAGGAGTCGCCGCCGTCCGT
CGAGATCTCCGTCTCCAAACCGCCGCCGCCACAACCGCCGCAGATCCATCACTTCGAGAA
CGGGAGCACCAGCACGCTCACGGAGAACCCCAGTCTCTCCGTGCACGCGCAGCAGCCTC
CGCCCCAGCAGGCGGCTGCGGCGGCGCAGAGGCAGAACCAGCACCAGCAGCAGCTCCA
GCATCAGCACCAGCTCCAGCTCCAGCACCAGCACAACCAGGGTCCTTTCCGCCGGGAGC
TCAATTTCTCAGATTTCGCGTCCAACGCATCCGTCACGGTGACCCCGCCTTTCTTCAAGCC
CGAGTCTGGTGAGATCCTAAACTTTGGCGCTGACAGCACCAGCCGGAGGAACCCTTCGCC
GGCGCCCCCCGCCGCGACGGCCAGCCTCACCACCGCGCCCGGGAGCCTATTCTCCCAG
CACACGGCGACTGTGACGGCCCCGTCAAACGACGCCAAGAACAACCCGAAGCGGTCCAT
GGAGGCCACCTCCCGCGCGAGCAACACCAACCACCACCAGAACGCCACAGCCAACGAGG
GGATGCTGTCCTTCTCGTCGGCGCCGACGACGCGGCCGTCCACCGGCACGGGCGCGCC
AGCCAAGTCGGAGTCCGACCACTCCGACCTGGAGGCGTCGGTCCGCGAGGTGGAGAGCA
GCCGCGTGGTGCCTCCGCCGGAGGAGAAGCGGCCGCGCAAGCGCGGGCGCAAGCCGG
CGAACGGGCGCGAGGAGCCCCTGAACCACGTGGAGGCGGAGCGGCAGCGGCGGGAGA
AGCTGAACCAGCGGTTCTACGCCCTCCGCGCCGTGGTGCCCAACGTGTCCAAGATGGAC
AAGGCCTCGCTGCTGGGCGACGCCATCTCCTACATCAACGAGCTCCGCGGCAAGATGAC
GGCGCTGGAGTCGGACAAGGAGACGCTCCACTCCCAAATCGAGGCGCTCAAGAAGGAGC
GCGACGCCCGGCCGGCCGCGCCGTCGTCGGGGATGCACGACAACGGGGCGCGGTGCC
ACGCGGTCGAGATCGAGGCCAAGATCCTGGGGCTGGAGGCGATGATCCGCGTGCAGTGC
CACAAGCGCAACCACCCGGCGGCGAAGCTGATGACGGCGCTGCGGGAGCTGGACCTGG
ACGTGTACCACGCCAGCGTGTCCGTGGTGAAGGACATCATGATCCAGCAGGTGGCGGTG
AAGATGGCCACCCGGGTCTACTCCCAGGACCAGCTCAACGCGGCGCTCTACGGCCGCCT
CGCCGAGCCGGGCACCGCGATGCAAATCCGGTAA-3’

Supplemental Figure 2. The coding sequence of *TaMYC4* from bread wheat cultivar KN199. The full length CDS of *TaMYC4* derived from the B subgenome is shown.

Supplemental Figure 3

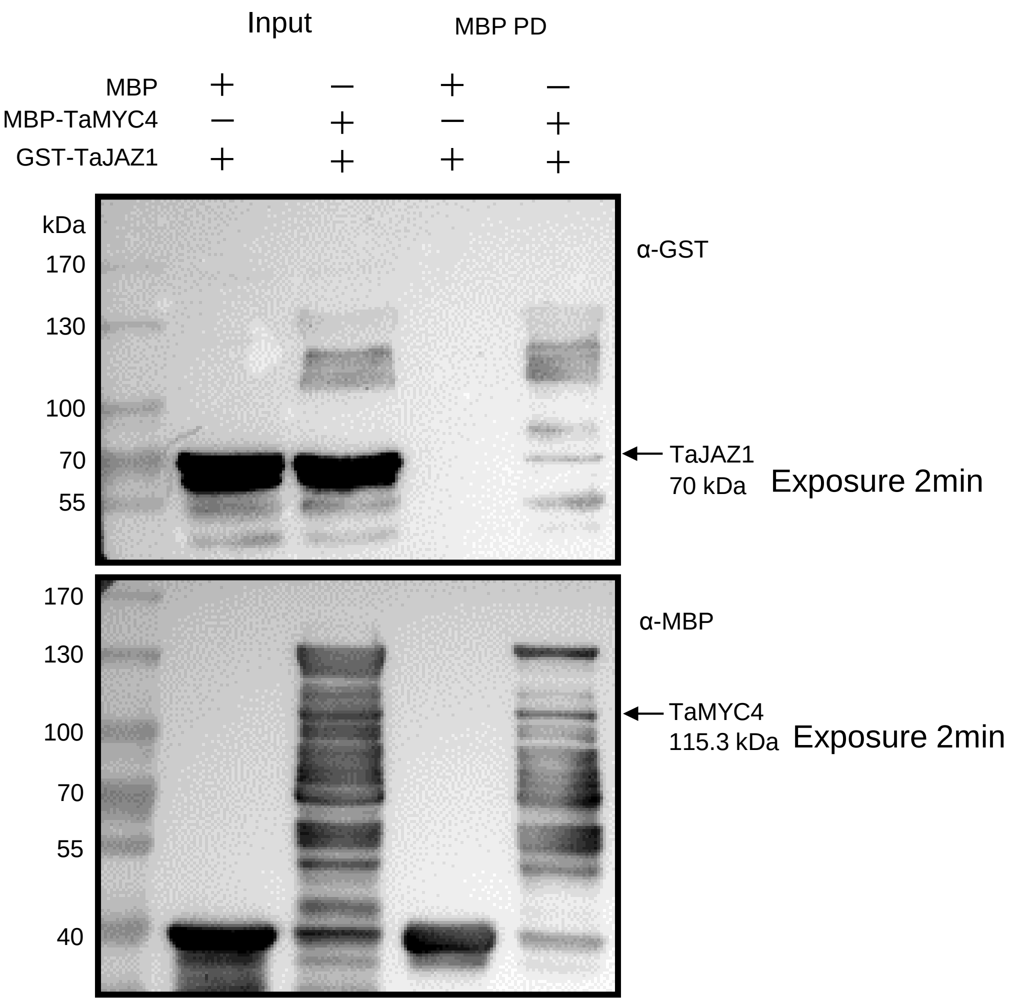

Supplemental Figure 3. Pull-down assay showing the interaction between TaJAZ1 and TaMYC4. GST-TaJAZ1 protein was pulled down by MBP-TaMYC4 protein, and detected using an anti-GST antibody. MBP and MBP-TaMYC4 were detected using anti-MBP antibody. Arrow indicates specific band.

Supplemental Figure 4

TaJAZ1-5A-promoter

5’-
TACGCTAAATGCTAAGCCGCTATCATCACGCCCTAATGCAACCGGGCGTCGCCACGTAGGGA
GACAAACCTGCATCATGTACAGTAATTAAGTACTGCTCCACTACGCAACCGATGAGCACCTTG
TTAAAGTAATCAACAGGGTCGATCAGCATGCTAACTGCTCCTCCTCCTAGTAGAGTGGCGTGG
AGTATACTATTGCTAATACTGCTAGCCGGTAGCTCCACATGGCCTGTACCTTATCTCCCTAGAT
TAACATTTAACAAAGTACTACTACTACTACTTAATCGTGGGATGCAGACGGGAATCTTGCACAG
TGTCTCGGACAAGTTTGACAAGACTCTGCTTGTATCACTAAATGATGTTATGGAAAGTACTCCA
TCTAGTCATCGTTTTACTTTTCATGGGTGCCAGTACAGTAGAGTATAGTCCATCACATAAAAAC
TTGGGAGATAAACAAAGCTATCTCAGTGTACCAAGTGATGCTACGAGTACCGTAGGAATTGAC
AAGAAGACGTGTTTGAATAGGAAACCATAATTTCGGGGCAAAAAGAGTTAACACACCATGATT
TGGTACTACTACCAGTAGTACTCCTATCCAATAGGTCACGGTCCGTGGTTCATGTTGATTTGAT
ATTTACTCCAAAGTGGACCATACAAAAAAACTCTCATTTTAAGCCGACATACAAAAGCTGTAAA
TTTGATCAAAAAGAGCACCTGAATGTTGCGTCGATAGATTCCTAAAAAATGCTACTCCACGAAG
AACAAATTCATTTGAAAATCCACGAAAGGCCCTCGAACTCGCCTCGAAAGGCCACGCACGGC
CGTCTCCTTTCCGAAAAGGCAACGTCACCATCTCTCTAATCTACTCACCGAACCTATAAAACTT
CGCTAAACAAATCCCGCAGCCTAAGCAAGGAATAGTACGTACAAGTCCAGCAGATGACCACAT
TAGCTCAAAAGAAAAAGCAGATGACCACATAGAGAAGAAAAAACAACATGGCGCGCACATGG
CACATGGTCATGGCCACAAGACATAAACAAAACCGGCGAAGCACGCCAGCCCCCCACCCCAC
CCCAACCCCAACCCCGTGGACGCAAATTTTTTAATCATCAGAAGACACACAAAAAAAGGAACA
TCACGGGATTAGTATTATTATTCTACCCATAAATCCAAAAGTCTACTGTAAGCTCGGGCGCCG
GGCCCCCCGCGACGCGCAGCACGTGGTCTTCCCCCGCGACCCACGCGCAGGGAATCCCGC
GGGCCCACAGCGTCAGCGACGGCCCGCTCCCCACCTGGTAATTCGGCTCGTGTGACCCAGC
CGGGGCTGCACGTGTCGCCACTGCAGCCGCCGCCAGGGCCCCTGTGCGCGGCTACCCGCA
CCGGGCGGCACTCACCGACGCGCGGGGCAGGGTCTGGGGTTTGACCCGGCTGTCAGTGGC
AGTGGGCGGCGTGGGAGCCTCGCGAGGCGGGATGCGCTCGTGCCCGACCACGTGGGCGC
GACCGGGCTGGGCCAGCGCGAGAGGAGAGGGCGCCCCGATCCGCTCACCGCGGTGGCCC
ACCGCCGTGCTCGCCACACCTTTCCTCTCGGTTGATTATCACTCGGTTTTGCTTGTTTATTTAT
CTATAGTTTGTTTATTTATTTAATTAATCAATCATCTGCCACCCGATCTAACCGCCTGGGCCGA
AAATAAATAAGCGATTAGCGGCACCACAAGCGCACGCACACCGCCTAGAGGCCATGCTTGGC
TAGGAGGAGTACTGGAGCAGTATACATACGAGAGAGAGAGAGGCAGGAAGGGAAAGGCAGA
GCGGTCAAGGCTGGGAGAGGGAGAGGGAGAGCATCGTCGTCGTCATCATTCGCTCGTTCAC
AGAGGAGTGAGAGAGTCAGAGGAGGAAGGAAGGTTCAGGGGAGTGAGAGACGAAATTTCC-3’

Supplemental Figure 4. Promoter sequence of *TaJAZ1* from bread wheat cultivar KN199.

Supplemental Figure 5

TaNINJA-1A

5’-
ATGGAGGATGGCCTTGAGCTTAGTTTAGGCCTCTCTTTGGGTGGTGGGTCTTCTGGGAAGT
CTAAGGCAAGAGATGCTTCTCTAGAGCCTAAAGCAGAACCTCAAGTGGAAGAGAGCAGTAG
CAAAGGCGTCTCGCAACCTCCCGATGCTCCCTTTGCGCATTACTATCAAGCAACTGCTGAGA
ACCAGGAACACAGTAGCAAGCAGAGGCATAGCCCTGCTACCCCGCCATTTGGAAACTTCTG
GGGACAACACGGTGGCTCCTCTGCTCCAGTGGCAGATGGATCAAGTGAACTAATAGCCCAC
CAATCTCAGCTTCCCCAGTATCAGGAGGGGCGGACTCCAAATAATATTGGAAATAATTCGGA
GGAAAACATGCCAGTCTCAAGTAAACGAAAGCTGCTTTCTGAAGAGACAAGTTTTCAGAAGA
AGCATCATTCTGCTGCTGACCAGCCTGATGCATTCAGTAAGAGCTCTGAGGGAGGTGTGAA
AAATGCACCGATTTCAATTAGCACGGATGATGGTTCAACAGGTGAAAATGAGGATGTTGCAG
ACTCGGAAGCAGAAGGCTCAAACTCTTGGTTGGTTGCACAGCGTGAAGACAGTGCCAAGGG
CTCTGTGGTAAACAGAGGATCCGATATAAAGAGATCCAGTGATGATGCTACAGGTGGATTTC
AAGGAAAAAGGCAACCGAGCTTTTCAGGAAGTGAATCTAGCTCTGGAAAGTTGCCGCATGG
AAATCCCTTACAAGCATCAAATGTAGTGACTGCACAATATCAAGGCCAGACTCAGGTTTCAG
CACCCCTGGGCATAACTAATGCACCCAATTTTCCTCCAATGTATACAGTGCAATTGAGGCCA
CCCGTAAACAATGGACCAGCGGTCCAGACGATGGGTGGTGCCTCCCAGGTTTCTTTTGGTT
ACCCAACAGGTCAGCTACCAATACTTGAAACGAGCTCTTCATGGGCATTTGGTACTCCGCCT
CAGGCTATTTCTTCCTTTGCTGCAAAGCAAGCCGAACGAGCAGGAGCCAAACAAGCTGATG
ATGGAAAGAAACCTCAAGAGGCTGGCGCGTCCTCCTCTGCTCTTTTGGAGGATGGTAAGGT
CGTTGAGAAGGTATTGCCTCTCATGGGCTCGGGCTCTGGTATAAGGCCGGGCATCGCCCC
GAATGTCAAATTCGGAGGGTCCGGATCATACCCTGATCTTCCCTGGGTATCCACAACCGGG
TCTGGACCAAATGGCAGGACCATATCGGGCGTAACATACAACTTCGGCAGAAACGAAGTGA
AGATCGTGTGTGCTTGCCATGGCACACACATGACGCCTGAGGAGTTCACGCGACACGCTAG
CGTGGACGCAACAGGCCAGGAGAACAATGCTACCATGTCGGCGTTTCCTGTCGGGAATCAA
GCGGCCTCAGCCCAAAACTAA-3’

Supplemental Figure 5. The coding sequence of TaNINJA from bread wheat cultivar KN199

Supplemental Figure 6

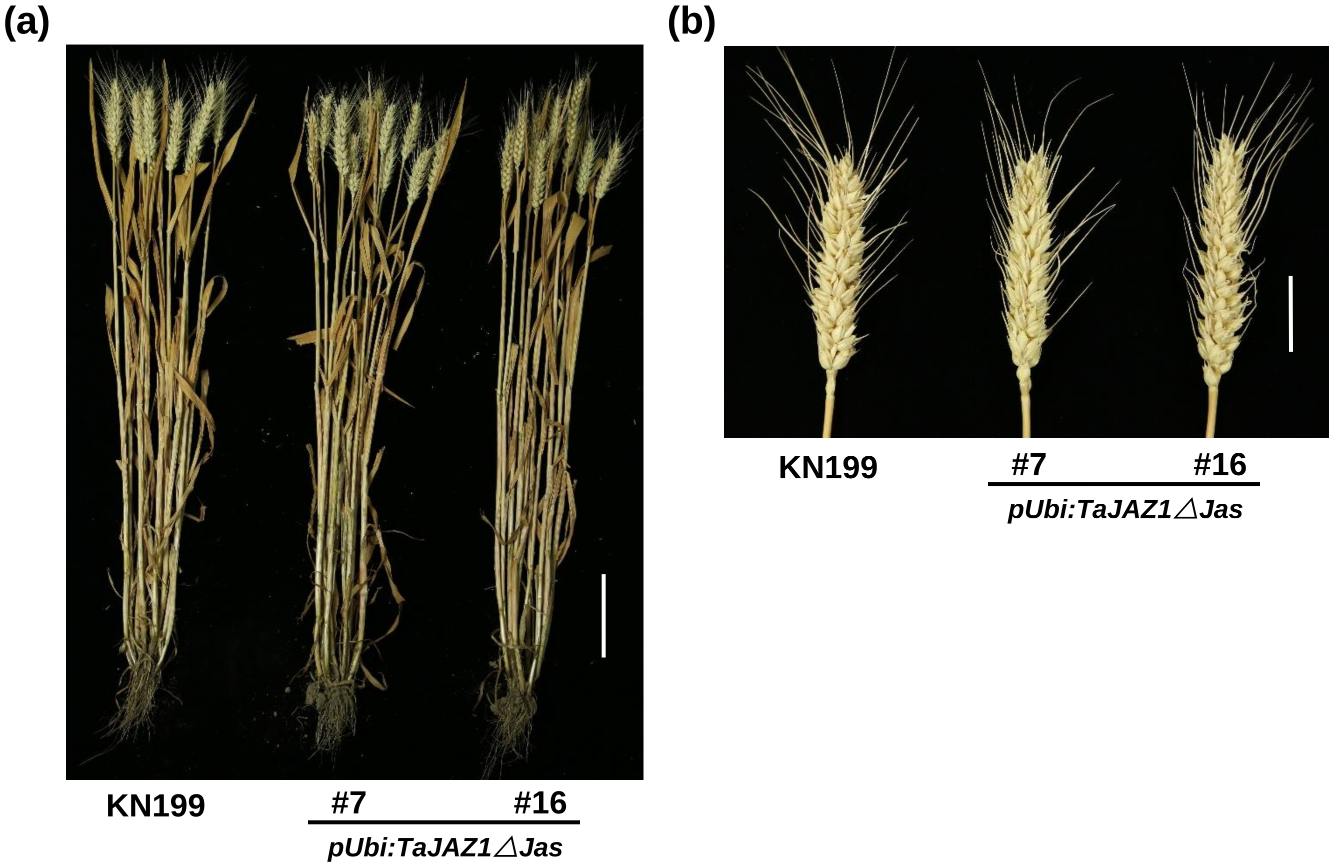

Supplemental Figure 6. Overexpression of TaJAZ1△Jas have no defects on plant growth phenotypes. (a) Appearance of a mature plant. Bar = 10 cm. (b) Spike morphology. Bar = 2 cm.

Supplemental Figure 7

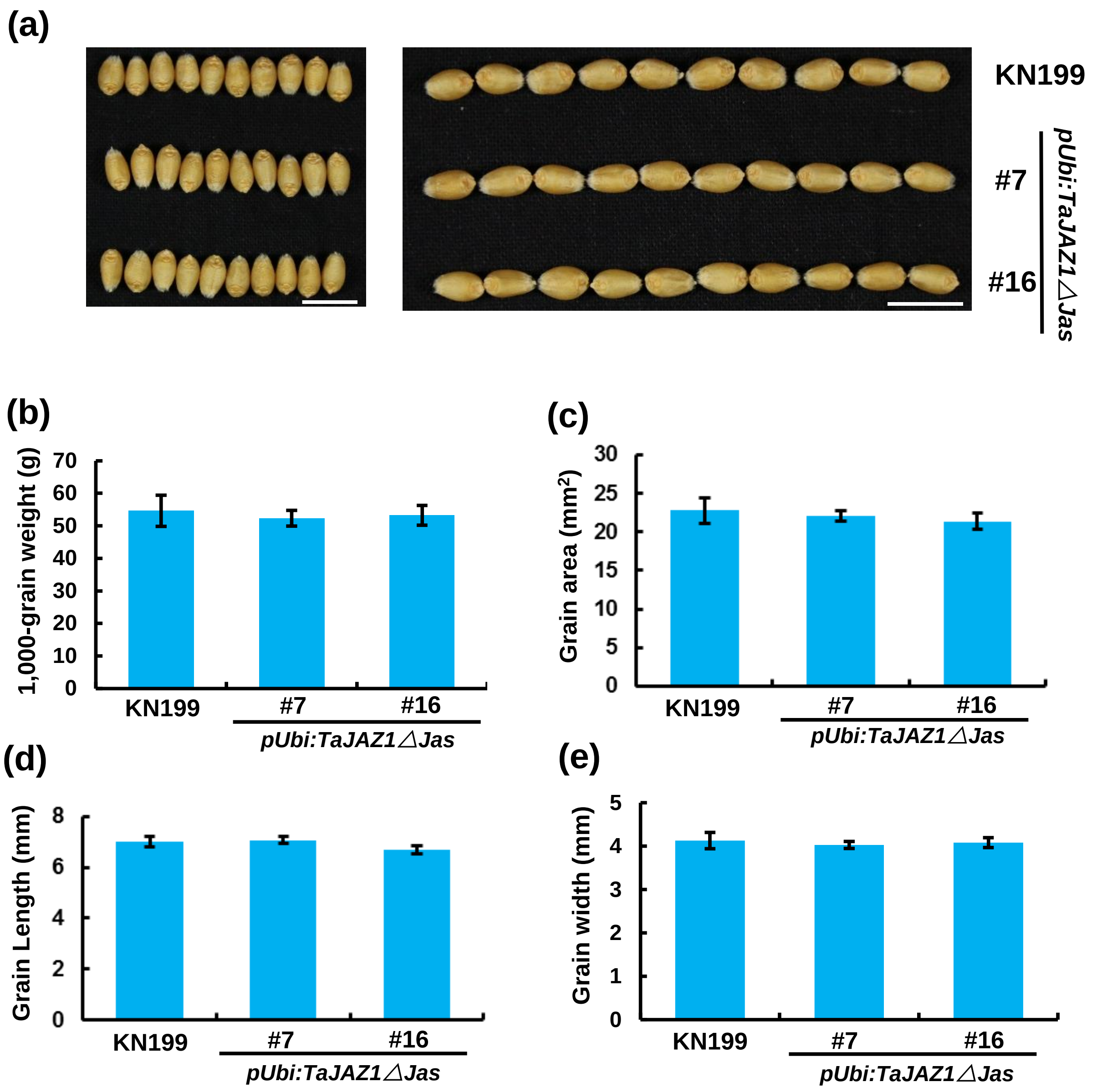

Supplemental Figure 7. Analysis of grain size in KN199 and two *pUbi:TaJAZ1△Jas* transgenic lines.
(a) Appearance of the grains in KN199 and transgenic plants. Comparisons of 1000-grain weight (b), grain area (c), grain length (d), grain width (e). Error bars denote ± SD (n = 6).

Supplemental Table 1

Supplemental Table 1. Constructs used in this study.

Construct name	Vector	Description
pUbi:TaJAZ1△jas	*pUbi:cas*	For gene tansformation
35S:TaJAZ1-GFP	*PGWB5*	For subcellular localization analysis For LUC activity assay
AD-TaJAZ1	*pGADT7*	For Y2H assay
BD-TaMYC4	*pGBKT7*	For transcriptional activity assay and Y2H assay
BD-TaMYC4-NT	*pGBKT7*	For transcriptional activity assay
BD-TaMYC4-CT	*pGBKT7*	For transcriptional activity assay
TaJAZ1pro:LUC	*pGWB35*	For LUC activity assay
TaMYC4-101	*pEarlyGate 101*	For LUC activity assay
MBP-TaMYC4	*pMAL-c2X*	For pull down assay
GST-TaJAZ1	*pGEX-4T-1*	For pull down assay
nYFP-TaJAZ1	*pEarly201*	For BiFC assay in *N. benthamiana*
cYFP-TaNINJA	*pEarly202*	For BiFC assay in *N. benthamiana*
nLUC-TaMYC4	*p1300-35S-nLUC*	For LCI assay in *N. benthamiana*
nLUC-TaMYC4-NT	*p1300-35S-cLUC*	For LCI assay in *N. benthamiana*
nLUC-TaMYC4-CT	*p1300-35S-nLUC*	For LCI assay in *N. benthamiana*
nLUC-TaNINJA	*p1300-35S-nLUC*	For LCI assay in *N. benthamiana*
nLUC-TaNINJA-NT	*p1300-35S-nLUC*	For LCI assay in *N. benthamiana*
nLUC-TaNINJA-MD	*p1300-35S-nLUC*	For LCI assay in *N. benthamiana*
nLUC-TaNINJA-CT	*p1300-35S-nLUC*	For LCI assay in *N. benthamiana*
cLUC-TaJAZ1	*p1300-35S-cLUC*	For LCI assay in *N. benthamiana*
cLUC-TaJAZ1-NT	*p1300-35S-cLUC*	For LCI assay in *N. benthamiana*
cLUC-TaJAZ1-CT	*p1300-35S-cLUC*	For LCI assay in *N. benthamiana*

Supplemental Table 2

Supplemental Table 2. Primers used in this study.

Primer name	Sequence (5'-3')
TaJAZ-B-F	AGGGGAGGGGAGGGGAGGGG
TaJAZ-B/D-F	CGAAATCCATTTATTCCCCGTC
TaJAZ-B/D-R	CCATAGATTCAACCATGATGGCTG
TaMYC4-outer-F	CTCCCATCCATTCCCCTTCC
TaMYC4-outer-R	CGATCATACGGTGGTGAGTAATAGG
TaMYC4-QBV3-F	ATGAACCTGTGGACGGACGA
TaMYC4-QBV3-R	CCGGATTTGCATCGCGGT
TaNINJA-outer-F	CCACCAGACTGCCTTGTGTTATAAT
TaNINJA-outer-R	ATATGGACACAAAGGGAACATCAAG
TaNINJA-QBV3-F	ATGGAGGATGGCCTTGAGCT
TaNINJA-QBV3-R	GTTTTGGGCTGAGGCCGC
TaTPL-outer-F	CAGGCTAGGTTTACGGCCAGG
TaTPL-outer-R	CTGAGATGGATACCTTGGTTAGTG
TaTPL-F	ATGTCTTCTCTCAGCCGGGAGC
TaTPL-R	TTATCTTTCTGGTTGATCAGAACTCGA
TaTPL-R(-stop)	TCTTTCTGGTTGATCAGAACTCGA
TaJAZ1-promoter-outer-F	CACAGAGAAGAAAGCACATGCAT
TaJAZ1-promoter-outer-R	AGTCCCTCTCCATCCAGCC
TaJAZ-promoter-QBV3-F	TACGCTAAATGCTAAGCCGCT
TaJAZ-promoter-QBV3-R	GGAATAAATGGATTTCGTCTCTCAC
TaJAZ-QBV3-F	ATGGAGAGGGACTTCCTGGG
TaJAZ-QBV3-R	GATGTGTAATTTTGTACTCGGGGAC
PUBI-BamH1-TaJAZ-F	CGGGATCCATGGAGAGGGACTTCCTGGGC
PUBI-Kpn1-TaJAZ-R	GGGGTACCTCAAACAGCTCTTGGCATAATACTTG
Real-TaJAZ1-F	GACACGCCGAAGCCAAAGAC
Real-TaJAZ1-R	GGCAAAGGAGGTGAAACACG
Real-TaMYC4-F	TTTCTCAGATTTCGCGTCCAAC
Real-TaMYC4-R	CTCGTTGATGTAGGAGATGGCG
Real-TaNINJA-F	CCTCAGGCTATTTCTTCCTTTGC
Real-TaNINJA-R	GGAAGATCAGGGTATGATCCGG
nLUC-TaMYC4-F	CACGGGGGACGAGCTCGGTACCATGAACCTGTGGACGGACGA
nLUC-TaMYC4-R	ACGCGTACGAGATCTGGTCGACCCGGATTTGCATCGCGGT
TaMYC4-N-Kpn1-F	GGGGTACCATGAACCTGTGGACGGACGA
TaMYC4-N-Sal1-R	GCGTCGACCGGCCGCTTCTCCTCC
TaMYC4-C-Kpn1-F	GGGGTACCATGCGCAAGCGCGGG
TaMYC4-C-Sal1-R	GCGTCGACCCGGATTTGCATCGCGG
TaJAZ1-N-Kpn1-F	GGGGTACCATGGAGAGGGACTTCCTGGG
TaJAZ1-N-Sal1-R	GCGTCGACAGATTTTGGCACTGCGGAAT
TaJAZ-C-Kpn1-F	GGGGTACCATGTCAGGCCAGTTGGTTGTG
TaJAZ-C-Sal1-R	GCGTCGACGATGTGTAATTTTGTACTCGGGGAC
nLUC-TaNINJA-F	CACGGGGGACGAGCTCGGTACCATGGAGGATGGCCTTGAGCT

Supplemental Table 2. Primers used in this study.

(Continued)

Primer name	Sequence (5'-3')
nLUC-TaNINJA-R	ACGCGTACGAGATCTGGTCGACGTTTTGGGCTGAGGCCGC
nLUC-TaNINJA-N-R	ACGCGTACGAGATCTGGTCGACAGCATCGGGAGGTTGCGA
nLUC-TaNINJA-M-F	CACGGGGGACGAGCTCGGTACCATGCCTTTTGCGCATTACTATCAA
nLUC-TaNINJA-M-R	ACGCGTACGAGATCTGGTCGACTTCGGCTTGCTTTGCAGC
nLUC-TaNINJA-C-F	CACGGGGGACGAGCTCGGTACCATGCGAGCAGGAGCCAAACAAG
TaJAZ1-4T-1-F	CCGCGTGGATCCCCGGAATTCATGGAGAGGGACTTCCTGGG
TaJAZ1-4T-1-R	ATCCGGCCGCTCGABTCGACTCAGATGTGTAATTTTGTACTCGGGGAC
TaMYC4-MBP-F	TTCAGAATTCGGATCCATGAACCTGTGGACGGACGA
TaMYC4-MBP-R	TTGCCTGCAGGTCGACTTACCGGATTTGCATCGCGGT
TaMYC4-N-BD-F	ATCTCAGAGGAGGACCTGCATATGATGAACCTGTGGACGGACGA
TaMYC4-N-BD-R	AGGTCGACGGATCCCCGGGAATTCCGGCCGCTTCTCCTCC
TaMYC4-C-BD-F	ATCTCAGAGGAGGACCTGCATATGATGCGCAAGCGCGGG
TaMYC4-C-BD-R	AGGTCGACGGATCCCCGGGAATTCCCGGATTTGCATCGCGG
TaMYC4-BD-F	ATCTCAGAGGAGGACCTGCATATGATGAACCTGTGGACGGACGA
TaMYC4-BD-R	AGGTCGACGGATCCCCGGGAATTCCCGGATTTGCATCGCGG
TaJAZ1-AD-F	CAGATTACGCTCATATGATGGAGAGGGACTTCCTGGG
TaJAZ1-AD-R	CACCCGGGTGGAATTCGATGTGTAATTTTGTACTCGGGGAC
Real-TaPR1-F	GAGAATGCAGACGCCCAAGC
Real-TaPR1-R	CTGGAGCTTGCAGTCGTTGATC
Real-TaPR2-F	AGGATGTTGCTTCCATGTTTGCCG
Real-TaPR2-R	AAGTAGATGCGCATGCCGTTGATG
Real-TaPOX2-F	AGGGGCTTCGGCGTCATC
Real-TaPOX2-R	TTGGGCGTCGTCGTGTCC
Real-TaSOD-F	CCGAGGTCTGGAACCATCAC
Real-TaSOD-R	AGCCGAAATCCTTCTCGATCT
